# Haplotype heterogeneity and low linkage disequilibrium reduce reliable prediction of genotypes for the ‑α ^3.7I^ form of α-thalassaemia using genome-wide microarray data

**DOI:** 10.12688/wellcomeopenres.16320.2

**Published:** 2021-09-22

**Authors:** Carolyne M. Ndila, Vysaul Nyirongo, Alexander W. Macharia, Anna E. Jeffreys, Kate Rowlands, Christina Hubbart, George B. J. Busby, Gavin Band, Rosalind M. Harding, Kirk A. Rockett, Thomas N. Williams, MalariaGEN Consortium

**Affiliations:** 1 Department of Epidemiology and Demography, KEMRI-Wellcome Trust Research Programme, Kilifi, PO BOX 230-80108, Kenya; 2 United Nation Statistics Division, United Nations, New York, New York, 10017, USA; 3 Wellcome Centre for Human Genetics, University of Oxford, Oxford, Oxfordshire, OX3 7BN, UK; 4 Centre for Genomics and Global Health, Big Data Institute, University of Oxford, Oxford, Oxfordshire, OX3 7LF, UK; 5 Parasites and Microbes Programme, Wellcome Sanger Institute, Wellcome Genome Campus, Hinxton, Cambridgeshire, CB10 1SA, UK; 6 Departments of Zoology and Statistics, University of Oxford, Oxford, Oxfordshire, OX1 3SZ, UK; 7 Department of Infectious Diseases, Imperial College Faculty of Medicine, London, W2 1NY, UK

**Keywords:** Malaria, α-thalassaemia, Predictive Models, multinomial regression-model, Classification and Regression Tree, GWAS, haplotypes

## Abstract

**Background: **The -α ^3.7I^-thalassaemia deletion is very common throughout Africa because it protects against malaria. When undertaking studies to investigate human genetic adaptations to malaria or other diseases, it is important to account for any confounding effects of α-thalassaemia to rule out spurious associations.

**Methods: **In this study, we have used direct α-thalassaemia genotyping to understand why GWAS data from a large malaria association study in Kilifi Kenya did not identify the α-thalassaemia signal. We then explored the potential use of a number of new approaches to using GWAS data for imputing α-thalassaemia as an alternative to direct genotyping by PCR.

**Results: **We found very low linkage-disequilibrium of the directly typed data with the GWAS SNP markers around α-thalassaemia and across the haemoglobin-alpha ( *HBA*) gene region, which along with a complex haplotype structure, could explain the lack of an association signal from the GWAS SNP data. Some indirect typing methods gave results that were in broad agreement with those derived from direct genotyping and could identify an association signal, but none were sufficiently accurate to allow correct interpretation compared with direct typing, leading to confusing or erroneous results.

**Conclusions: **We conclude that going forwards, direct typing methods such as PCR will still be required to account for α-thalassaemia in GWAS studies.

## Introduction

With recent advances in high-throughput GWAS technologies, a growing number of studies are now being conducted with a view to investigating the contribution of genetics to the risk of a broad range of human diseases. However, such single-nucleotide polymorphism (SNP)-based approaches are imperfect because they only capture a limited picture of total genetic diversity. Specifically, while many SNPs and other short variants across the genome are now routinely accessed by these studies, other important pathogenic variants including larger insertions, deletions and other structural rearrangements are not typically assayed directly. Some of these, such as the thalassaemia-causing mutations of the α-globin ( *HBA1-HBA2)* gene region ^1^, cannot be accurately imputed from current reference panels ^2, 3^, raising a circle of questions about the mutational origin, ancestry and haplotype structure of these variants.

**Figure 1.**
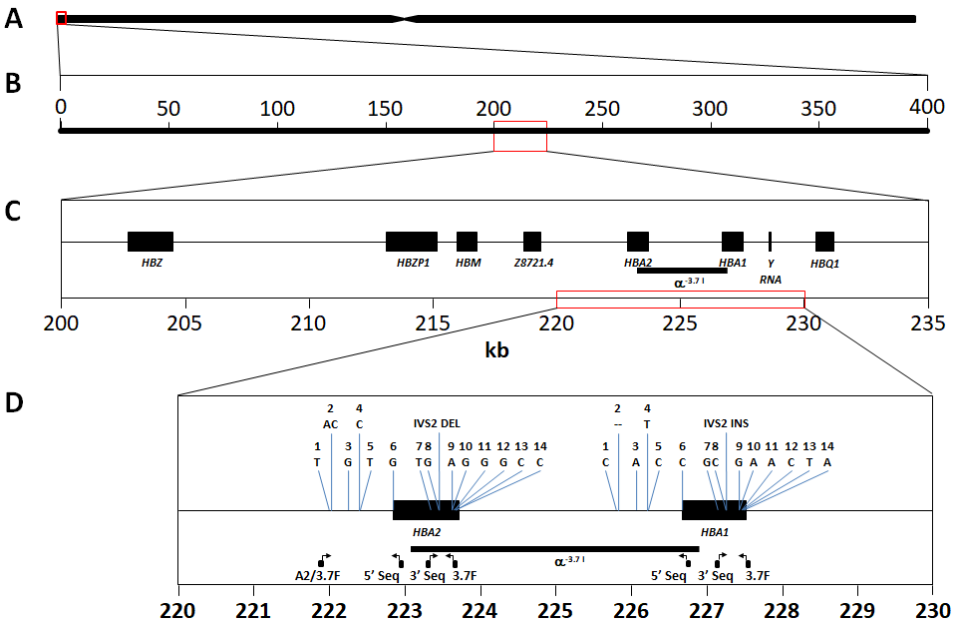
Schematic of the *HBA* region on human chromosome 16. **A**: Representation of human chromosome 16 showing the location of the *HBA* gene region at the p-telomere end (red box). **B**: The 400kb chromosome region spanned by the SNPs used in this study (16:83,000-400,000) approximately centres around the *HBA* gene region (red box). **C**: Chromosome 16:200000-235000 spanning the classical *HBA* gene region, comprising *HBZ* [ζ2], *HBZP1* [ψζ1], *HBM* [ψα1], *HBA2* [α2], *HBA1* [α1] and *HBQ1* [θ1]. The -α ^-3.7I^ deletion is highlighted between *HBA1* and *HBA2*. **D**: *HBA1* and *HBA2* genes showing the location of the primers used for genotyping and Sanger sequencing (see methods and Extended Data); the region of the -α ^-3.7I^ deletion; and 15 bases/features that show paralogous differences in the human reference genome between *HBA1* and *HBA2* sequences and used to identify the -α ^-3.7^ Type I breakpoint (Extended Data).

The α-thalassaemias are among the commonest known genetic conditions in humans ^4^. They have probably arisen because of an elevated *de novo* mutation rate ^5, 6^ coupled with the fact that they confer a survival advantage against death from *Plasmodium falciparum* malaria ^5– 7^. As a group, the α-thalassaemias are characterized by the reduced or absent production of the essential α-globin component of normal haemoglobin ^4^. α-globin is encoded by a pair of adjacent genes ( *HBA1* and *HBA2*) that lie within the haemoglobin-alpha ( *HBA*) gene locus on chromosome 16 ( Figure 1) ^8^. Although many genetic forms of α-thalassaemia have been described worldwide ^8^, to the best of our knowledge, the 3.7kb Type I deletion (-α ^3.7I^) is the only pathogenic variant that occurs at significant frequencies throughout most of Africa ^9^. This variant appears to result from a cross-over event between homologous intergenic regions within the *HBA* gene cluster which gave rise to a hybrid gene consisting of the 5’ part of *HBA2* and the 3’ part of *HBA1* ^10^. Although α-globin production is reduced in affected subjects, enough is still synthesized that clinically, both heterozygotes (-α/αα) and homozygotes (-α/-α) are essentially normal ^4^, while haematologically they display only marginal anaemia and reduced red blood cell volumes ^11^. Despite its high allele frequencies and potential importance as a confounder in GWAS studies in African populations, the -α ^3.7I^ deletion is not well-captured using regional SNP-based genotype inference methods ^2, 3^. Notwithstanding this, recovering α-thalassaemia genotypes from GWAS data could be very useful given the amount of data now available worldwide, particularly as it would be difficult or impossible to genotype such samples retrospectively for α-thalassaemia.

In this current study we use α-thalassaemia genotype data from a previous study of more than 6,000 children from Kilifi, Kenya ^12^, along with their corresponding Illumina HumanOmni2.5-4 microarray data ^3^, to investigate the haplotype structure surrounding the α-thalassaemia deletion variants in this population, and to gain an insight into why inferred α-thalassaemia genotypes were not well captured. We also explored the potential utility of a wide range of other indirect GWAS-based approaches, including the microarray-chip intensity data and haplotype imputation, as an alternative to direct typing of α-thalassaemia, which is technically challenging and adds additional costs.

## Methods

### Study population

Participants were recruits to a case-control study of severe malaria as described in detail previously ^12^. Briefly, cases were children presenting to Kilifi County Hospital in Kenya with features of severe falciparum malaria, while controls were children recruited from the surrounding community to a genetic cohort study of childhood illness (the Kilifi Genetic Birth Cohort Study ^13^). A subset of 3,036 participants ( Table 1) were selected from this study for whom GWAS data and α-thalassaemia sickle (rs334) genotypes were already available from previous studies ^3, 12^.

**Table 1.** Main characteristics of the studied population and distribution of the chromosomes used in the analysis.

Characteristics	Overall	Genotyped at the α-thalassaemia locus		
		Homozygous ancestral (αα/αα)	Heterozygous derived (-α/αα)	Homozygous derived (-α/-α)
**All subjects, N (%)**	**3036**	**1139 (37.5)**	**1474 (48.6)**	**423 (13.9)**
**Cases**	**1432 (47.2)**	**588 (19.4)**	**673 (22.2)**	**171 (5.6)**
**Controls**	**1604 (52.8)**	**551 (18.1)**	**801 (26.4)**	**252 (8.3)**
**Gender, N (%)**				
**Males**	**1543 (50.8)**	**579 (19.1)**	**750 (24.7)**	**214 (7.0)**
**Females**	**1493 (49.2)**	**560 (18.4)**	**724 (23.8)**	**209 (6.9)**
**Ethnicity, N (%)**				
**Giriama**	**1494 (49.2)**	**547 (18.0)**	**715 (23.6)**	**232 (7.7)**
**Chonyi**	**946 (31.2)**	**342 (11.3)**	**482 (15.9)**	**122 (4.0)**
**Kauma**	**271 (8.9)**	**114 (3.8)**	**124 (4.1)**	**33 (1.1)**
**Others (18)**	**325 (10.7)**	**136 (4.5)**	**153 (5.0)**	**36 (1.2)**
**Thalassaemia** **chromosomes, N**				
**αα**	**3752**	**1139**	**1474**	**-**
**-α**	**2320**	**-**	**1474**	**423**
**Sickle (rs334)** chromosomes *, N (%)				
**AA**	**2730 (89.9)**	**1026 (33.8)**	**1317 (43.4)**	**387 (12.7)**
**AT**	**283 (9.3)**	**104 (3.4)**	**146 (4.8)**	**33 (1.1)**
**TT**	**23 (0.8)**	**9 (0.3)**	**11 (0.4)**	**3 (0.1)**

Numbers given are for all individuals included in this study, with percentages shown in parentheses by row. * The A allele encodes for normal β-globin while the T allele encodes for β ^s^-globin such that AT individuals have sickle cell trait and TT individuals have sickle cell disease.

### Ethics approval and consent to participate

The study was approved by the Kenya Medical Research Institute/National Ethical Review Committee in Nairobi, Kenya (Number: SCC1192), and by the Oxford Tropical Research Ethics Committee in Oxford UK (Number: OXTREC 020-06). Written informed consent for inclusion in this study was given by all participants or their parents.

### Sample genotyping

We used data derived using the Illumina HumanOmni2.5-4 genotyping chip (Illumina, California, USA), which were aligned to Human Genome build GRCh37 and curated as previously described ^3, 14, 15^ and that are already publicly available ^16^. Details of how to access the GWAS data package can be found on the MalariaGEN website and in the Data Availability section below. For the analysis of the α-globin region we extracted genotype data and intensity data from the chromosome 16 vcf file (Extended Data Figure 1; Kenya_GWAS-2.5M_b37_chr16_aligned.vcf.gz) into GEN format using QCtool, (options and parameters are shown in Extended Data Figure 1), to which we included lists of excluded samples (Extended Data Figure 1; Kenya_GWAS-2.5M_b37.sample [762/3869 samples]) and SNPs (Extended Data Figure 1; Kenya_GWAS-2.5M_b37_snp_qc.txt [21756/80392 SNPs]) based on information in the QC files provided with the Kenya GWAS dataset package (sample and SNP missingness of ≤ 0.05, allele frequencies of ≥ 0.01 and filtration for curated sample and SNP duplicates) and our own sample requirements (data on clinical status [GWAS data package], data for both α-thalassaemia [Underlying Data2 Table AA_sample_codings], rs334 genotyping [GWAS data package] and gender [GWAS data package]; Extended Data Figure 1; alphathal_sickle_genotypes.csv; Kenya_all_samples.sample; clin_phenotypes.csv). The QCtool outputted a 0-10Mb region of chromosome 16 giving 9723 SNPs for 3107 samples (Extended Data Figure 1). At this point the α-thalassaemia data were merged into the data set using QCtool and then phased into the haplotypes with ShapeIT v2 (options and parameters are shown in Extended Data Figure 1). An African recombination map (Underlying Data2 Table CC_chr16_recombination) was included for this step. Following phasing, the sample set was reduced by a further 71 samples missing HbS (sickle) genotype and/or gender data (used as important covariates in our analysis). After this phasing step, we selected all polymorphisms across the 400kb region from the p-arm telomere (spanning 84870 – 398421bp) of chromosome 16 spanning *HBA2* and *HBA1* where the -α ^3.7 ^deletion is located (219454–227532: Underlying Data1 and Underlying Data2 Table BB_0-400kb_snp_details). This resulted in a final dataset comprising 179 polymorphisms (178 SNPs and the -α ^3.7I^ deletion by direct typing) (Underlying Data2 Table BB_0-400kb_snp_details) for 6072 chromosomes from 3036 individuals ( Table 1) ^12, 17^.

### Association analysis

All association analyses were performed using R (Extended Data Table 13) as previously described ^12^. The sample size for this study was determined pragmatically, based on the number of samples that were available from our previous study in Kenya ^3, 15^, and the completeness of the GWAS data available ( Table 1). Odds ratios for SNP associations with severe malaria were determined by comparison of allele and genotype frequencies among cases and controls, using a fixed-effects logistic-regression model, both with (Underlying Data2 Table JJ_assoc_results_adjusted_hbs) and without (Underlying Data2 Table II_assoc_results_unadjusted) adjustment for the confounding effects of genetic background (using self-reported ethnicity) and rs334 genotype, the SNP responsible for both sickle-cell trait (HbAS) and sickle-cell disease (HbSS), all of which are major potential confounders in the interpretation of such analyses. Gender is also associated with malaria risk both at a genetic and at a social/cultural level, and was also included as a co-variate in our adjusted analyses.

### Linkage disequilibrium (LD) and haplotype frequency estimation

We determined genotype frequencies for α-thalassaemia and all SNPs individually by direct allele counting (Underlying Data2 Table BB_0-400kb_snp_details). Pairwise LDs were estimated by calculating r, r ^2^ and D' metrics using a custom R script (Extended Data Section 7; Underlying Data2 Tables KK_pairwise_R, LL_pairwise_R2 and MM_pairwise_Dprime). Extended haplotype homozygosity (EHH) and bifurcation diagrams were calculated in R using *rehh* (Extended Data Table 13). Haplotype tree structures were analyzed using the R packages *hclust* and *dendrograms* (Extended Data Table 13) and were visualized using *pegas* and *dendextend* (Extended Data Table 13). Haplotype diversity ^18^ was estimated using with *hap.div* function within *pegas*.

### Sanger sequencing to confirm the form of α-thalassaemia present in the Kilifi population.

**Figure 2.**
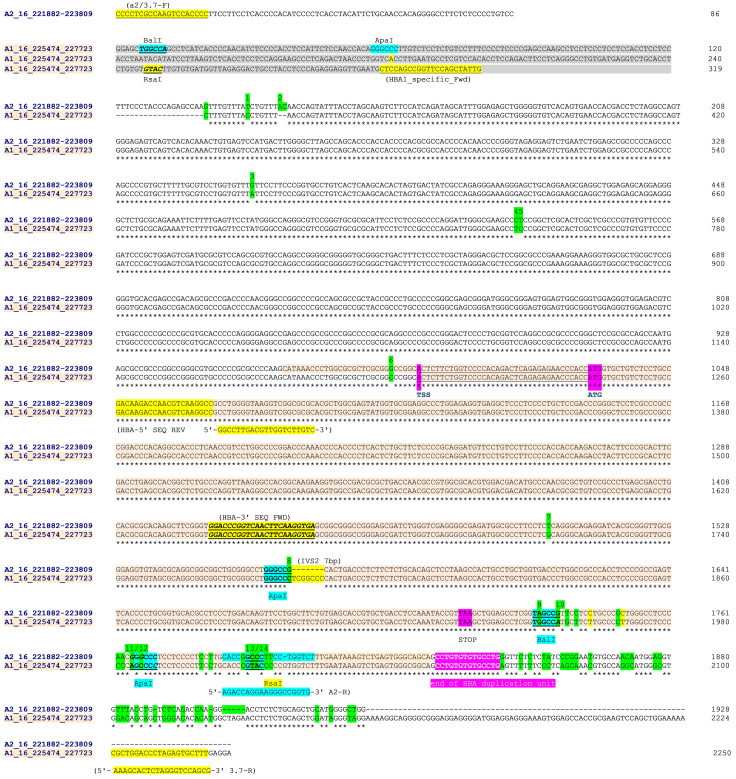
Sequence alignment from Clustal Omega across *HBA2* and *HBA1* on human chromosome 16 (Sequence data from Ensemble GRCH37). Sequences were aligned in chromosomal order ( *HBA2* [prefix 'A2_16_'] above *HBA1* [prefix 'A1_16_']). The *HBA2* sequences starts at the 5'-most base of the forward PCR primer (A2/3.7-F position 16:221882) in a unique region and 86 bases 5' of the homologous region with *HBA1*. The *HBA1* sequence starts at position 16:225474 which is 319 bases from the equivalent homologous region with *HBA2*. The *HBA2* sequence ends at position 16:223809 effectively at the end of the homologous region with *HBA1*, while the *HBA1* sequences ends at position 16:22773 and the PCR reverse primer (3.7-R). PCR and sequencing primers ( Table 2) are highlighted (A2/3.7-R,HBA-5'-SEQ-REV, HBA-3'SEQ-FWD and 3.7-R) as are key restriction sites ^10^. Paralogous differences between *HBA1* and *HBA2* reference sequences are highlighted in green for a set of 14 positions. These were used to help identify the -α ^3.7^ deletion type from Sanger sequencing. The *HBA* genic region is coloured and shows the transcription start site (TSS), amino-terminal methionine (ATG) and stop codons (TAA); but not separate introns and exons for clarity. The four restriction sites used to distinguish the -α ^3.7^ deletion types are identified ^10^; the Type I breakpoint was identified as being 5' of the ApaI/IVS2 sequence; the Type II breakpoint lies between the ApaI/IVS2 and BalI restriction sites; The Type III lies between the RsaI and the 'end of *HBA* duplication unit' (location and identity of *HBA* duplication unit ^27^). NB: The ApaI/IVS2 sequence comparison between *HBA2* and *HBA1* has been aligned here to clearly highlight the ApaI restriction site; it may be shown differently in other publications.

**Figure 3.**
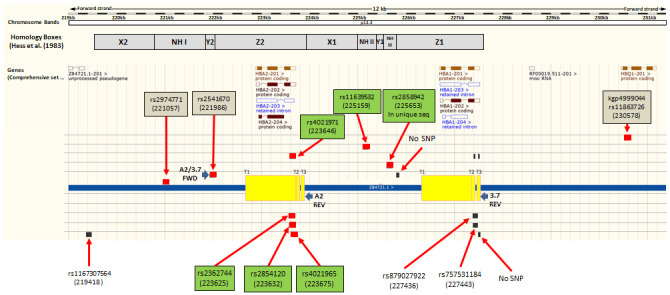
Map of the *HBA* region on human chromosome 16 identifying Illumina chip features flanking and internal to the α ^-3.7^kb deletion. Ensembl GRCh37 chromosome 16 *HBA* region; Illumina HumanOmni2.5-4 feature match; (red boxes are perfect match of probe with ref sequence; Black boxes are lesser matches; boxes on same row/level are from same probe); SNP name boxes have GRCh37 positions; Green boxes are six features within the deletion region while the three brown boxes are the SNPs immediately flanking the region. Non-highlighted labels are other blast hits. Yellow boxes show regions of breakpoints/crossovers for the three known types of -α ^3.7^kb deletion; Homology boxes X, Y and Z indicated as per Hess *et al*. ^28^.

Genotyping of the α-thalassaemia polymorphism was undertaken as described ^19^ and sequences (forward orientation) for the human reference sequences of the *HBA2/HBA1* (16:221882-227718) region were downloaded from Ensembl GRCh37 to span the forward and reverse PCR primers used to determine the presence or absence of the -α ^3.7^-deletion ( Table 2). The *HBA2* region spanning the common forward PCR primer (A2/3.7F; 16:221882-221901 [ Table 2], that lies at the beginning of the Y2 box, to approximately 50bp 3' of end of the X-box [16:223809] [ Figure 3]) was aligned with an equivalent *HBA1* region between 16:225474 and the *HBA1* reverse PCR primer (3.7-R; 16:227698-227718 [ Table 2, Figure 2]). Clustal Omega was used to generate the alignment and manual finishing was used to finalise any misaligned regions and identify features ( Figure 2). From the aligned sequences, a number of key differences were identified across the homologous *HBA2* and *HBA1* regions that included seven individual bases, the 7bp IVS2 INDEL and four restriction sites (ApaI, BalI, ApaI and RsaI – 1, 2, 3, 2 base-differences, respectively ^10^) at the 3' end of the *HBA* coding region ( Figure 2 and Table 3 [Underlying Data2 Table NN_Sanger_Sequence_summary]).

**Table 2.** Primer details for Sanger sequencing the alpha-thalassaemia deletion region in *HBA1* and *HBA2*.

Name	Dir	Genome Position	Gene	Genome Position	Genome Position	Primer Sequence (5'-3')
A2/3.7-F	FWD	16:221882-221901	5' to *HBA2*			CCCCTCGCCAAGTCCACCC
*HBA1*_specific_F	FWD			16:225771-225792	*HBA1*	CTCCAGCCGGTTCCAGCTATTG
*HBA*-5' SEQ REV	REV	16:222930-222950	*HBA2*	16:226734-226754	*HBA1*	GGCCTTGACGTTGGTCTTGTC
*HBA*-3' SEQ FWD	FWD	16:223310-223332	*HBA2*	16:227114-227136	*HBA1*	GGACCCGGTCAACTTCAAGGTGA
A2-R	REV	16:223666-223684	3' to *HBA2*			AAAGCACTCTAGGGTCCAGCG
3.7-R	REV			16:227698-227718	3' to *HBA1*	CCCCTCGCCAAGTCCACCC

α-thalassaemia PCR was undertaken using the standard protocol as described ^19^. PCR products were then sequenced using the primers above (see methods). A2/3.7-F, A2-R and 3.7-R are primers used in the standard PCR assay for α ^-3.7^-thalassaemia deletion. *HBA*-5' SEQ REV and *HBA*-3' SEQ FWD were designed internal to the products to allow more coverage. All primer locations are identified in Figure 2

For Sanger sequencing, 25 individuals identified as homozygous for the -α ^3.7^ deletion (using primers A2/3.7F and 3.7-R [hybrid *HBA2-HBA1*]) and 9 individuals homozygous wild-type (primers A2/3.7F and A2-R [ *HBA2*], and *HBA1*_specific_F with A3.7R [ *HBA1*]) were selected and used to generate fresh PCR product as previously described ^19^. We used the same set of 9 wild-type individuals for sequencing both *HBA1* and *HBA2*.

Amplicon presence and genotype classes were confirmed by running an aliquot of the PCR product by agarose gel electrophoresis. The remaining PCR product was cleaned using the Qiagen PCR clean-up kit (QIAquick PCR purification kit, Qiagen, Crawley, UK) and quantified using picogreen (Quant-iT ^TM^, Thermo Fisher Scientific, Loughborough, UK). Amplicons and sequencing primers ( Table 2) were prepared according to instructions and sent to Eurofins (Eurofins Genomics UK, Wolverhampton, UK) for processing. In summary;

- PCR amplicons for *HBA2* were generated from 9 individuals who were identified as wild-type for *HBA1* and *HBA2* using primers A2/3.7-F, and A-2R ( Table 2).
- PCR amplicons for *HBA1* were generated from 9 individuals who were identified as wild-type for *HBA1* and *HBA2* using primers *HBA1*_specific_-F, and A3.7R ( Table 2) – NB: these were the same individuals as for the *HBA2* sequencing.
- PCR amplicons for the -α ^3.7^ deletion were generated from 25 individuals who were identified as homozygotes using primers A2/3.7-F and 3.7-R ( Table 2).
- Sanger sequencing was undertaken on all amplicons using their respective forward and reverse PCR primers, plus 2 internal primers; HBA-5'.SEQ and HBA-3' ( Table 2).

Sequence traces for individuals' samples were inspected and curated using Chromas to generate FASTA files for alignment on Clustal Omega with the human reference sequences (Extended Data Sections 9-12 for pile-ups). Alignments were then manually finished before inspection and the key paralogous bases in the human reference sequence that distinguish *HBA2* from *HBA1* ( Figure 2) were identified and recorded ( Table 3 [Underlying Data2 Table NN_Sanger_Sequence_summary]).

**Table 3.** Sanger sequencing of PCR amplicons for the α ^-3.7^kb deletion in 25 homozygous α ^-3.7^kb and 5 normal individuals in Kilifi, Kenya.

																	Released_key		648184022	836466359	891210787	539560608	391787794	604702281	818082173	251883490	640859222	130037624	787645827	167368322	114762936	115163414	116814170	116828578	123017600	129122739	130028961	130546094	131709234	134168958	134421137	134501405	137928280	520269712	434039943	424597966	429643240	754351794	429586481	117865639	297748035	138218129	520269712	434039943	424597966	429643240	754351794	429586481	117865639	297748035	138218129
																	Chip_id														5583612155_R01C01	5583612121_R04C01	5583612103_R04C01	5583612084_R03C01	5583612106_R04C01	5583612069_R01C01	5588392045_R04C01	5583612069_R03C01	5583612132_R02C01	5588392041_R02C01	5588392009_R02C01	5767745121_R02C01	5626627008_R01C01	5751823150_R01C01	5636411175_R03C01	5636622086_R02C01	5626627093_R01C01	5636622089_R03C01	5636411079_R02C01	5636411197_R03C01	5636622060_R04C01		5751823150_R01C01	5636411175_R03C01	5636622086_R02C01	5626627093_R01C01	5636622089_R03C01	5636411079_R02C01	5636411197_R03C01	5636622060_R04C01	
Sequencing primers					note	Base-gene assignment	Base ID No.	HBA2				HBA1				Base ID No	Reference allele																																												
A2/3.7-F	HBA 5' SEQ	HBA 3' SEQ	3.7-R	A2-R				position	base	rs number	relative to ATG	position	base	rs number	relative to ATG		HBA2	HBA1																																											
					primer (A2/3.7-F)			221882-901	CCCCTCGCCAAGTCCACCCC	NA		NA	NA	NA	NA				**1**	**2**	**3**	**4**	**5**	**6**	**7**	**8**	**9**	**10**	**11**	**12**	**13**	**14**	**15**	**16**	**17**	**18**	**19**	**20**	**21**	**22**	**23**	**24**	**25**	**A**	**B**	**C**	**D**	**E**	**F**	**G**	**H**	**I**	**A**	**B**	**C**	**D**	**E**	**F**	**G**	**H**	**I**
						T2/C1	1	221996	T	rs1043385709	-916	225802	C	NA	-916	1	**T**	**C**	**T**	**T**	**T**	**T**	**-**	**T**	**T**	**T**	**-**	**-**	**-**	**-**	**-**	**-**	**-**	**-**	**-**	**-**	**-**	**-**	**-**	**-**	**-**	**-**	**-**	**T**	**T**	**T**	**T**	**T**	**T**	**T**	**T**	**T**	**-**	**C**	**C**	**-**	**C**	**C**	**-**	**C**	**C**
						AC2/--1	2	222003-004	AC	rs1388103544	-908	225808-809	del	rs1297040092	-908	2	**AC**	**--**	**AC**	**AC**	**AC**	**AC**	**-**	**-**	**AC**	**AC**	**-**	**-**	**-**	**-**	**-**	**-**	**-**	**-**	**-**	**-**	**-**	**-**	**-**	**-**	**-**	**-**	**-**	**AC**	**AC**	**AC**	**AC**	**AC**	**AC**	**AC**	**AC**	**AC**	**-**	**--**	**--**	**-**	**--**	**--**	**-**	**--**	**--**
						G2/A1	3	222239	G	rs143196637	-673	226043	A	rs3760045	-673	3	**G**	**A**	**G**	**G**	**G**	**G**	**G**	**G**	**G**	**G**	**G**	**G**	**G**	**G**	**-**	**G**	**G**	**G**	**G**	**G**	**G**	**G**	**G**	**G**	**G**	**G**	**G**	**G**	**G**	**G**	**G**	**G**	**G**	**G**	**G**	**G**	**-**	**G**	**A**	**-**	**A**	**G**	**A**	**A**	**G**
						CT2/TC1	4	222416	CT	rs2858943	-496	226219	TC	rs878891332	-496	4	**C**	**T**	**T**	**T**	**T**	**T**	**T**	**T**	**T**	**T**	**T**	**T**	**T**	**T**	**T**	**T**	**T**	**T**	**T**	**T**	**T**	**T**	**T**	**T**	**T**	**T**	**T**	**T**	**T**	**T**	**T**	**T**	**T**	**T**	**T**	**T**	**-**	**T**	**T**	**-**	**T**	**T**	**T**	**T**	**T**
						CT2/TC1	5	222417	C T	rs4021962	-495	226220	T C	NA	-495	5	**T**	**C**	**C**	**C**	**C**	**C**	**C**	**C**	**C**	**C**	**C**	**C**	**C**	**C**	**C**	**C**	**C**	**C**	**C**	**C**	**C**	**C**	**C**	**C**	**C**	**C**	**C**	**C**	**C**	**C**	**C**	**C**	**C**	**C**	**C**	**C**	**-**	**C**	**C**	**-**	**C**	**C**	**C**	**C**	**C**
						G2/C1	6	222846	G	NA	-43	226679	C	NA	-43	6	**G**	**C**	**C**	**C**	**-**	**-**	**C**	**-**	**-**	**C**	**C**	**C**	**C**	**C**	**C**	**C**	**C**	**C**	**C**	**C**	**C**	**C**	**C**	**C**	**C**	**C**	**C**	**C**	**C**	**C**	**C**	**C**	**C**	**C**	**C**	**C**	**-**	**C**	**C**	**-**	**C**	**C**	**C**	**C**	**C**
					Ensembl Gene start			222864	NA	NA	-37	226673	C	NA	-37																																														
					Met start (ATG)			222912-914	ATG	NA	1	226716	ATG	NA	1																																														
					primer (HBA 5' SEQ)			222930-950	GACAAGACCAACGTCAAGGCC	NA	18-38	226734-754	GACAAGACCAACGTCAAGGCC	NA	18-38																																														
					primer (HBA 3' SEQ)			223310-332	GGACCCGGTCAACTTCAAGGTGA	NA	399-401	227114-136	GGACCCGGTCAACTTCAAGGTGA	NA	399-401																																														
						T2/G1	7	223383	T	rs2362746	471	227187	G	rs148228241	471	7	**T**	**G**	**G**	**G**	**K**	**K**	**G**	**K**	**G**	**G**	**K**	**K**	**K**	**G**	**G**	**G**	**G**	**G**	**K**	**K**	**G**	**G**	**K**	**K**	**G**	**G**	**K**	**T**	**T**	**T**	**T**	**T**	**T**	**T**	**T**	**T**	**-**	**G**	**G**	**-**	**G**	**G**	**G**	**G**	**G**
					ApaI	G2/C1	8	223447	G	NA	535	227251	C	NA	535	8	**G**	**C**	**C**	**C**	**C**	**C**	**C**	**C**	**C**	**C**	**C**	**C**	**C**	**C**	**C**	**C**	**C**	**C**	**C**	**C**	**C**	**C**	**C**	**C**	**C**	**C**	**C**	**G**	**G**	**G**	**G**	**G**	**G**	**G**	**G**	**G**	**-**	**C**	**C**	**-**	**C**	**C**	**C**	**C**	**C**
					(IVS2)	del 2/ins 1	IVS2	223447-448	del	NA	534	227252-258	TCGGCCC	NA	534	IVS2	**DEL**	**INS**	**INS**	**INS**	**INS**	**INS**	**INS**	**INS**	**INS**	**INS**	**INS**	**INS**	**INS**	**INS**	**INS**	**INS**	**INS**	**INS**	**INS**	**INS**	**INS**	**INS**	**INS**	**INS**	**INS**	**INS**	**INS**	**DEL**	**DEL**	**DEL**	**DEL**	**DEL**	**DEL**	**DEL**	**DEL**	**DEL**	**-**	**INS**	**INS**	**-**	**INS**	**INS**	**INS**	**INS**	**INS**
					STOP codon (TAA)			223597-599	TAA	NA	685	227408-410	TAA	NA																																															
					BalI	2 NOT-CUT/1 CUT	9	223614	T AGCCG	rs2362745	702	227425	T GGCCA	rs1170850212	702	9	**A**	**G**	**G**	**G**	**G**	**G**	**G**	**G**	**G**	**G**	**G**	**G**	**G**	**G**	**G**	**G**	**G**	**G**	**G**	**G**	**G**	**G**	**G**	**G**	**G**	**G**	**G**	**-**	**A**	**A**	**A**	**A**	**A**	**-**	**A**	**A**	**-**	**G**	**G**	**-**	**G**	**G**	**G**	**G**	**G**
					BalI		10	223618	TAGCC G	rs3209623	706	227429	TGGCC A	rs376827361	706	10	**G**	**A**	**A**	**A**	**A**	**A**	**A**	**A**	**A**	**A**	**A**	**A**	**A**	**A**	**A**	**A**	**A**	**A**	**A**	**A**	**A**	**A**	**A**	**A**	**A**	**A**	**A**	**-**	**G**	**G**	**G**	**G**	**G**	**-**	**G**	**G**	**-**	**A**	**A**	**-**	**A**	**A**	**A**	**A**	**A**
					ApaI	2 CUT/1 NOT-CUT	11	223646	GGGCCC	rs4021971	734	227424	AGCCCC	rs1463287368	734	11	**G**	**A**	**A**	**A**	**A**	**A**	**A**	**A**	**A**	**A**	**A**	**A**	**A**	**A**	**A**	**A**	**A**	**A**	**A**	**A**	**A**	**A**	**A**	**A**	**A**	**A**	**A**	**-**	**G**	**G**	**G**	**G**	**G**	**-**	**G**	**G**	**-**	**A**	**A**	**-**	**A**	**A**	**A**	**A**	**A**
					ApaI		12	223648	GG GCCC	rs4021970	736	227426	AG CCCC	rs1190061123	736	12	**G**	**C**	**C**	**C**	**C**	**C**	**C**	**C**	**C**	**C**	**C**	**C**	**C**	**C**	**C**	**C**	**C**	**C**	**C**	**C**	**C**	**C**	**C**	**C**	**C**	**C**	**C**	**-**	**G**	**G**	**G**	**G**	**G**	**-**	**G**	**G**	**-**	**C**	**C**	**-**	**C**	**C**	**C**	**C**	**C**
					primer (A2-R)			223666-683	AGACCAGGAAGGGCCGGTG	NA	-	NA	NA	NA	NA																																														
					RsaI	2 NOT-CUT/1 CUT	13	223672	G CCC	rs4021966	760	227483	G TAC	NA	760	13	**C**	**T**	**T**	**T**	**T**	**T**	**T**	**T**	**T**	**T**	**T**	**T**	**T**	**T**	**T**	**T**	**T**	**T**	**T**	**T**	**T**	**T**	**T**	**T**	**T**	**T**	**T**	**-**	**-**	**-**	**-**	**-**	**C**	**-**	**C**	**C**	**-**	**T**	**T**	**-**	**T**	**T**	**T**	**T**	**T**
					RsaI		14	223673	GC CC	rs1487591924	761	227484	GT AC	NA	761	14	**C**	**A**	**A**	**A**	**A**	**A**	**A**	**A**	**A**	**A**	**A**	**A**	**A**	**A**	**A**	**A**	**A**	**A**	**A**	**A**	**A**	**A**	**A**	**A**	**A**	**A**	**A**	**-**	**-**	**-**	**-**	**-**	**C**	**-**	**C**	**C**	**-**	**A**	**A**	**-**	**A**	**A**	**A**	**A**	**A**
					Ensemble gene end			223709	NA	NA	NA	227521	NA	NA	NA																																														
					primer (3.7-R)			NA	NA	NA	844-864	227698-718	CGCTGGACCCTAGAGTGCTTT	NA	844-864																																														

Individuals were identified following PCR typing for the -α ^3.7^ deletion and selected for Sanger sequencing of the amplicons. From the sequence traces the identity of a set of differentiating bases was recorded (*Base ID No.) as highlighted in Figure 2. The table rows show features ordered by chromosome position. Sequencing primers: These columns show the span of the Sanger sequencing typically covered by the named primers. Note: field intifies specific features across the *HBA2* and *HBA1* region; primers; gene start, stop; restriction enzyme sites ^10^ distinguishing the -α ^3.7^ types I, II and III. *HBA2*, *HBA1*: these columns identify the chromosomal position, sequence, rsIDs and position relative to the ATG site for *HBA2* and *HBA1* aligned to each other. Sequenced individuals are shown with their unique IDs (chip_id and released keys that match them to the main datasets). For individuals, the bases identified are shown and coloured according to the reference sequence (green – *HBA1*, orange – *HBA2*). Bases in purple were found to be identical across all individuals sequenced rather than divergent as in the reference sequences. Dashes are bases that were not possible to identify due to short sequences. The full table of this data can be found in Underlying Data2 Table NN_Sanger_Sequence_summary.

### Illumina chip intensity data

The Illumina genotyping method creates intensity data at two channels (X and Y), one for each of the alleles, for each feature (typically a SNP) ^20^. We identified all features on the Illumina chip used for genotyping the samples (HumanOmni2.5-4v1_H) across the -α ^3.7^ deletion region that sits between the Z-boxes of *HBA2* and *HBA1* [16:219454-227532] (Underying Data2 Tables BB_0-400kb_snp_details and OO_HBA_region_features, Figure 3), and extracted intensity data for these SNPs across all samples. We also included intensity data for the 5' and 3' SNPs immediately flanking the deletion region that were used in the haplotype analysis (rs2974771 [16:221057] and rs9936930 [16:233272]; Extended Data Sections 4 and 5). Because the intensity data may not always be normally distributed and the group sizes between genotypes may not be even, we made comparisons between genotype intensities for each SNP using the Kruskal-Wallis non-parametric test followed by Dunn’s test to compare each pair of genotypes ^21, 22^. We also computed Cohen's *d* and Hedges’ *g* effect size metrics ^23– 25^. (Underlying Data2 Tables PP and QQ).

### Inferring α-thalassaemia genotypes from intensity data

Given that we know the location and breakpoints for the α-thalassaemia deletion under investigation, we have looked at the SNPs and intensities directly to determine in an *a priori* fashion how well they infer the genotypes for the -α ^3.7^ deletion. We note that other predicative/inference methods are available in addition to those we have used, some designed to identify/discover non-SNP features using intensity data. Accordingly, we have provided all the information and data necessary for others to test alternative methodologies if interested.

***A) Direct analysis of intensity distribution***

**Figure 4.**
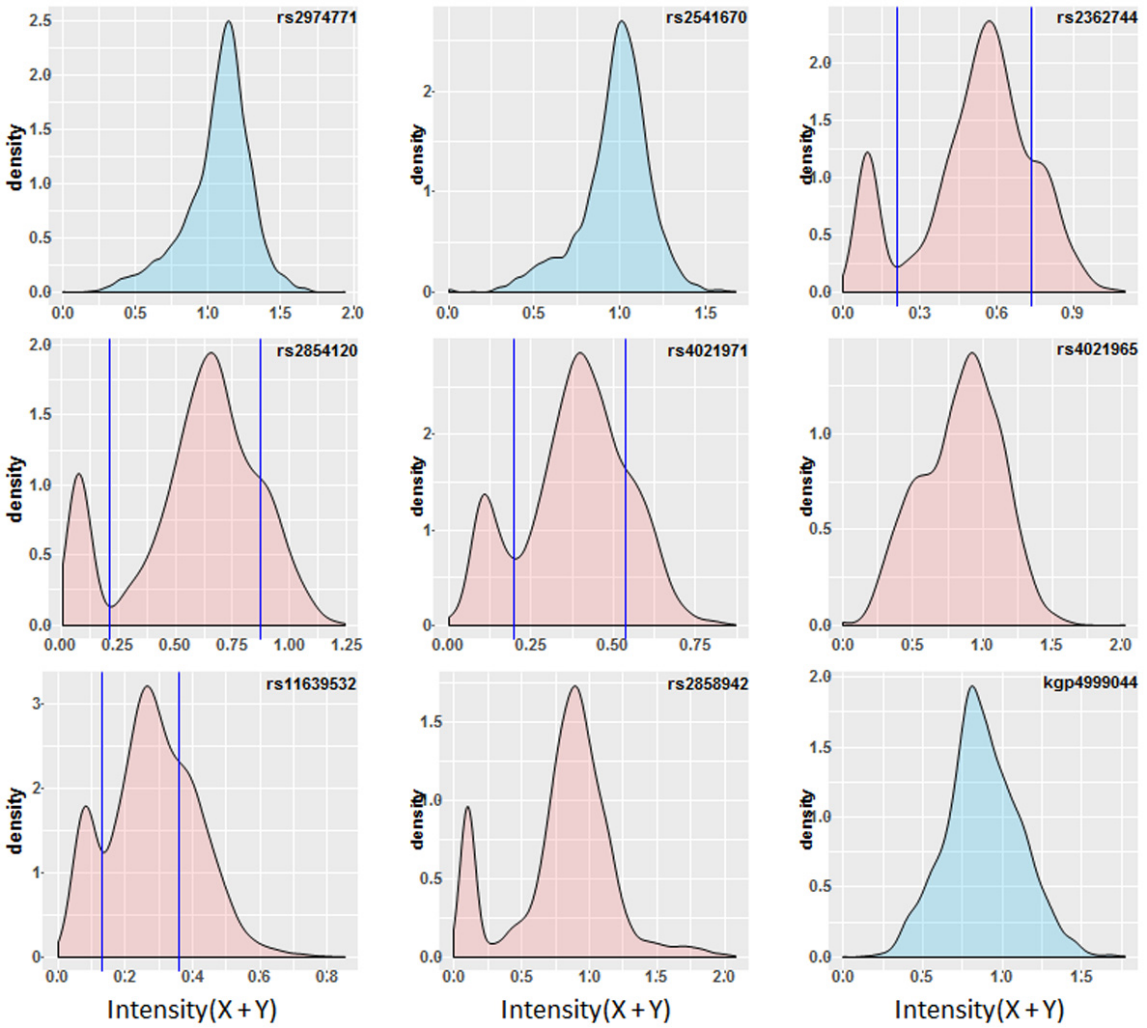
Chip Intensity (sum[X and Y] channels) density plots for features internal and immediately flanking the α ^-3.7^ deletion. Features filled in blue are flanking to the deletion (rs2972771 and kgp4999044 are present in the haplotype SNP data, rs2541670 did not pass QC). Features filled in red are within the -α ^3.7^ deletion. Vertical lines illustrate where both a trough and shoulder are discernible in the distribution and potentially infer the breaks between genotypic groups; rs2362744 0.211 and 0.738; rs2854120 0.215 and 0.869; rs4021971 0.199 and 0.538; rs11639532 0.131 and 0.358.

The first was based directly on the intensity data while the second involved models built on small amounts of data derived by direct genotyping. In the first instance for each of the six chip features located in the deletion region we plotted the distribution of the sum of the X and Y channel intensities as a density function and then selected two clear troughs or shoulders (C1, C2) in the density distributions ( Figure 4). Genotypes were then assigned as; homozygous for the derived/deletion allele <= C1; C1 < heterozygous < C2; and homozygous for the ancestral allele >= C2 (Extended Data Sections 4a and 4b).

***B) Hierarchical clustering of intensities***

**Figure 5.**
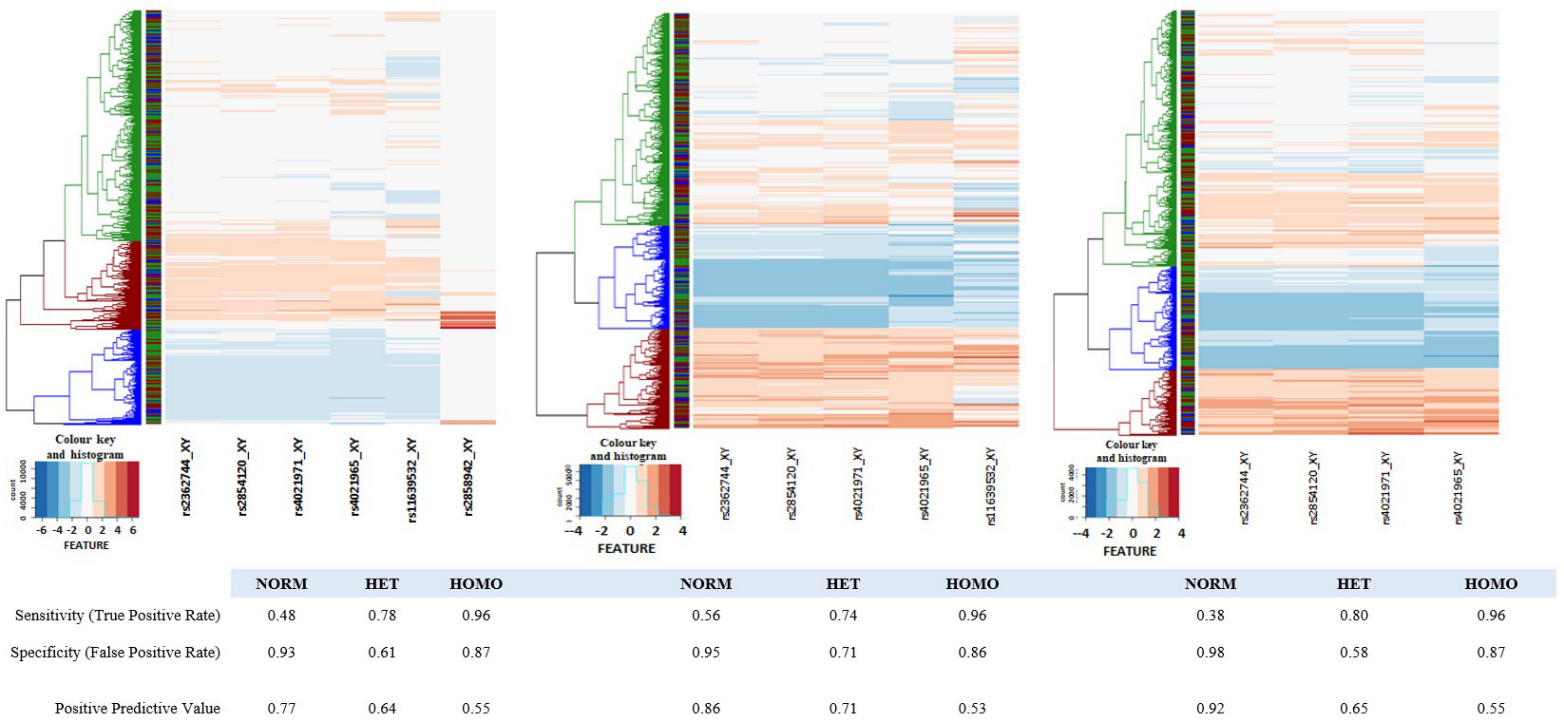
Heatmaps of core deletion feature intensities. Heatmaps were generated for four, five and six SNP features inside the -α ^3.7I^ deletion region. Sample-intensities were clustered as shown by the dendrograms at the left side of each panel and genotypes assigned (Blue: Homo, Green: Het, Red: Norm). In each case the intensities between SNPs were normalised to create a common intensity profile (Colour key and Histogram inset at the bottom left of each panel). Left-hand panel: all six SNP-features; Middle panel: five SNP-features with rs2858942 removed; Right-hand panel: four SNP-features with rs2858942 and rs11639532 removed. The ribbon between the dendrogram and the SNP features show the directly-typed genotype assignments in the same colour scheme.

We next undertook a hierarchical clustering of the data for the six chip features located in the -α ^3.7I^ deletion. This was applied using the *heatmap2* function of the *gplots* package in R ^28^. We created heatmaps for all six deletion features ( Figure 5) but as can be seen in panel A, the 2 3'-most features have different intensity profiles to the other four features. We have therefore also created heatmaps for the 5 5'-most features and the 4 5'-most features. In both the latter cases we identified the three most likely clusters corresponding to the three genotype classes (red, αα/αα normal; green, -α/αα heterozygotes; blue, -α/-α homozygotes). The assigned genotypes were extracted and compared with the directly-typed genotypes (Underlying Data2 Table TT_del_hier_cluster_assignment).

***C) Multiple-Regression Model (MRM) and Classification and Regression Trees (CART)***

a) MRM

The MRM extends the binary logistic models when there are more than two outcome categories. This approach was used to model, classify and predict multi-category outcomes conditional on a given set of explanatory variables. For an individual case (denoted as *i*), we want to predict whether the α-thalassaemia genotype is homozygous for -α ^3.7I^ (homo), heterozygous (het) or homozygous wild-type (norm) given intensities of predictor SNPs rs236744, rs2854120, rs4021971, rs4021965, rs11639532, rs2858942.

Let the tuple $X={\left[x_{i}^{k},y_{i}^{k}\right]}_{k=1}^{3}$ denote the intensity values for the *i ^th^* individual at genotype *k* = 1,2,3 (het, homo, norm respectively) for each SNP. As in other forms of linear regression, the MRM uses a linear function of chosen predictor variables, say:

$$l(T=t_{g}|X)=a_{g}+\sum_{k}\left(b_{gk}x_{i}^{k}+c_{gk}y_{i}^{k}+d_{gk}x_{i}^{k}y_{i}^{k}\right)\;equation 1$$

Where

$$Homo_{i}=a_{1}+b_{1}x_{i}^{1}+c_{1}x_{i}^{2}+d_{1}x_{i}^{3}+e_{1}y_{i}^{1}+f_{1}y_{i}^{2}+g_{1}y_{i}^{3}+h_{1}x_{i}^{1}y_{i}^{1}+m_{1}x_{i}^{2}y_{i}^{2}+n_{1}x_{i}^{3}y_{i}^{3}\;equation 2$$

*T* = *t _g_* and denotes α-thalassaemia het, homo or norm genotypes ( *g* = 1,2,3 respectively); *a _g_*, *b _gk_*, *c _gk_* and *d _gk_* are regression coefficients that are learned from a training dataset (a set of samples with known genotypes).

Note that *l*( *t _g_* | *X*) is a function of learned parameters and intensities that we can use to predict unknown α-thalassaemia genotypes wherever the intensities of each SNP are known. Thus, for given intensities of *X*, and parameters *a _g_*, *b _gk_*, *c _gk_* and *d _gk_*, the probability that α-thalassaemia genotypes *T* = *t _g_* are het, homo or norm for the *i ^th^* individual are:

$$\text{a})\;p(t=t_{1})=\frac{1}{1+\exp(l(t_{2},X))+\exp(l(t_{3},X))}i^{th}\;\text{heterozygote}\;equation 3$$

$$\text{b})\;p(t=t_{2})=\frac{\exp(l(t_{2},X))}{1+\exp(l(t_{2},X))+\exp(l(t_{3},X))}i^{th}\;-\alpha^{3.7}\;\text{homozygote}\;equation 4$$

$$\text{c})\;p(t=t_{3})=\frac{\exp(l(t_{3},X))}{1+\exp(l(t_{2},X))+\exp(l(t_{3},X))}i^{th}\;\text{wild-type}\;equation 5$$

i. MRM construction

**Figure 6.**
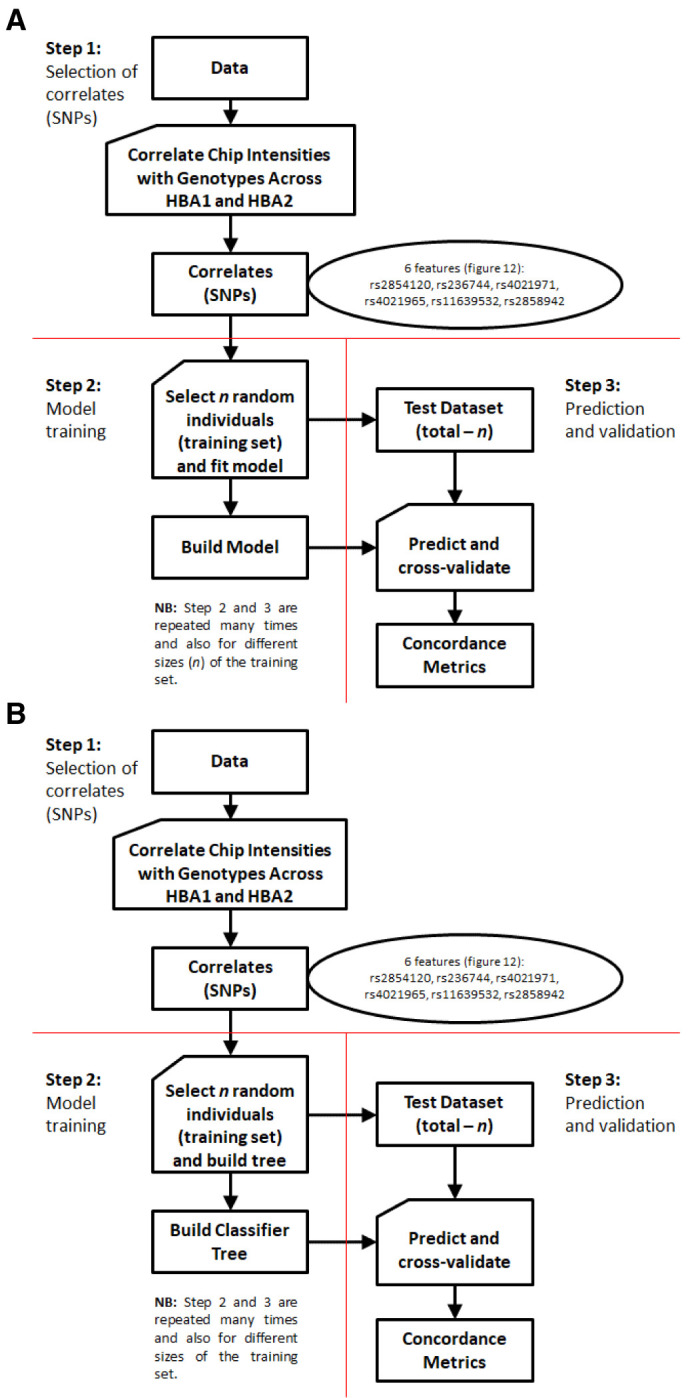
Multiple-Regression Model (MRM) and Classification And Regression Tree (CART) process flows. **A**: MRM process flow. This is a simple extension of binary logistic regression that allows for more than two categories of the dependent or outcome variable. The model can then be applied to new explanatory variables (i.e. without known genotypes) to predict unknown genotypes **B**: CART process flow. This method builds a binary decision tree (i.e. a series of evaluations based on a single concomitant variable at each point) and aims to split the data such that there is maximal separation of individuals in terms of the variable of interest. At each point, the evaluation of an individual is either positive or negative and the procedure seeks a cut-off point for a range of values of the concomitant variable such that the positive and negative groups contain maximal number of individuals of the same type. These learned series of evaluations can then be applied to a new set of individuals with concomitant variables known (without known types) to predict their unknown types. Here the concomitant variables are the intensities while the “types” are genotypes.

A general process flow is shown in Figure 6A. We first selected the 6 SNP-features located in the -α ^3.7I^ deletion region (rs236744, rs2854120, rs4021971, rs4021965, rs11639532, rs2858942) and extracted the intensity data from the Illumina chips. We then made a random selection of *n*=50 to *n*=500 individuals with known α-thalassaemia genotypes (the training set) for each of the SNP-features. These were used to calculate a set of parameters that the model could use to predict genotypes in the remaining (3036 – *n*) individuals. Finally, using these parameters the model assigned a probability to each α-thalassaemia genotype category for each individual. The category with the highest predicted probability was used to define the genotype for that individual. We repeated this 1000 times for each set of *n* samples to calculate a mean and SD and a predictive power score for each genotype class using the directly typed results as baseline. From the plots of the performances (Extended Data Figure 27A) we were able to identify a minimum training set of *n* = 100 individuals that could be used for further analysis using ROC curves and association analysis (Example run: Underlying Data3).

b) CART

i. Background

CART is a machine-learning approach that has been shown to be particularly useful when analyzing non-linear relationships and it is thought to be more robust than the standard regression models for classification ^29^. Furthermore, the approach is simple to understand or interpret and requires little data preparation. It uses “decision trees” to classify new data.

ii. CART Model construction

A general process flow is shown in Figure 6B. We first selected the 6 SNP-features located in the -α ^3.7I^ deletion region (rs236744, rs2854120, rs4021971, rs4021965, rs11639532, rs2858942) and extracted the intensity data from the Illumina chips. We then made a random selection of *n*=50 to *n*=500 individuals with known α-thalassaemia genotypes (the training set) for each of the SNP-features. These were used to calculate a set of parameters that the model could use to predict genotypes in the remaining (3036 – *n*) individuals.

We used CART to build a binary tree by subdividing the data at each possible split and choosing the best split that produces the most homogenous sub-groups (as an example Extended Data Figure 26 shows the CART dendrogram and cut-offs applied to the polar cluster plots for each SNP for the full dataset). The predictive variables (the chip feature intensities) included in the model were the same as the MRM approach. The process of growing the tree was stopped when the number of individuals within each node was less than five. We repeated this 1000 times for each set of *n* (50 – 500) samples to calculate a mean and SD and a predictive power score for each genotype class using the directly typed results as baseline. From the plots of the performances (Extended Data Figure 27B) we were able to identify a minimum training set of *n* = 100 individuals that could be used for further analysis using ROC curves and association analysis (Example run: Underlying Data3).

***D) Imputation using haplotypes***

An increasingly popular way to infer missing genotype information is by statistical imputation using haplotype structure. This method employs a reference set of haplotypes (such as the 1000 Genomes resource ^30– 32^) for comparison with the observed test data generated on genome-wide chip arrays. The IMPUTE2 program ^33, 34^, was developed to phase the test data (if not already done so) and compare to the reference data set to find the most probable haplotype matches to infer (impute) any missing polymorphic sites in the data.

Our dataset comprised 178 polymorphisms directly typed on the Illumina HumanOmni2.5-4 for the 0-400kb region on chromosome 16 (see above and Extended Data Section 1) in 3036 individuals with the addition of PCR-directly-typed α-thalassaemia genotypes (see above and Extended Data Section 1) for comparison testing.

IMPUTE2 requires a reference set of haplotypes to infer missing genotypes. However, we decided not to use the 1000 genome reference panel for several reasons;

- The 1000G dataset does not contain any directly typed α-thalassaemia deletion variants, only imputed from the sequence data.
- The 1000G dataset does not have any populations that directly match the populations used in our study (the closest is a Western Kenyan population [LWK – Luhya]).
- There is also evidence that the 1000G imputed α-thalassaemia genotypes are not very reliable ^3^.

Other reference panels are becoming more widely available, some of which also include additional populations of African origin. While we decided not to use these for similar reasons to those described above for the 1000 Genomes data, we do not rule out the possibility that some could prove more suitable for imputing haplotypes into Kenyan populations.

Instead, we used our own data for creating both a reference dataset and for imputing missing genotypes, as this should have provided the best opportunity to predict/impute the α-thalassaemia genotypes. This entailed using a random sampling and bootstrap methodology (similar to the MRM and CART methods above). For a given run of IMPUTE2 and in line with the 1000 genomes population sizes (and the outcome of the MRM and CART methodologies above) we randomly selected 100 individuals (200 chromosomes) from our sample set to use as the reference set (known haplotypes [option *–h*]). These data were formatted in accordance with IMPUTE2 file policy. The remaining 2936 individuals' data had the α-thalassaemia data removed and identified as missing. These data were then saved in the appropriate IMPUTE2 format (option *–known_haps_g* given our data were already phased [Extended Data Section 1]). Other files required were; a legend file (option – *l*) for the polymorphisms in the dataset (SNP information file); a recombination map (option *-m*) which was taken from the African recombination map as described above and in Extended Data Section 1); a strand file (option *_strand_g*) identifying the orientation of each SNP (i.e. forward or reverse strand; all have already been orientated to forward); an interval for inference (option *–int* our region does not require 'chunking' and can be used in its entirety); an effective population size (option *–Ne*) for which we have used the default setting of 20,000 since we are not using the HapMap or 1000G populations.

An imputation run was then triggered using the following command line:



            impute2
     -m <recombination map file> 
     -h <reference haplotype set – 200 chr (100 individuals)> 
     -l <legend file>
     -known_haps_g <haplotypes with missing data – 5872 chr (2936 individuals)>
     -strand_g <strand file>
     -int 84870 398421
     -Ne 20000 
     -o <output file> 
          


Once complete, the output files were inspected and the α-thalassaemia imputation genotype probabilities extracted from the *.output* file. We also stored the directly-typed genotypes of the samples alongside each imputation run.

We undertook 1000 runs of IMPUTE2 using the above method. Upon completion of each run, the data were processed to call α-thalassaemia genotypes for each sample based on the IMPUTE2 probabilities using two thresholds (relaxed; 0.7 and strict; 0.9) to allow direct comparison to the typed genotypes. A thousand ‘runs’ were made and the performance was aggregated across all runs. We measured no-call rates and concordance rates from each of the runs. We also performed association testing, not only for the imputed data but for corresponding set of directly typed data; using the latter to account for variation in the reduced dataset (Extended Data Section 6).

## Results

We used genome-wide data derived from the Illumina HumanOmni2.5-4 genotyping array (Illumina, California, USA), that was collected within a case-control study conducted on the coast of Kenya as described in detail previously ^3, 12^. The current study included data from 3,036 of the children from the above study (1,432 severe malaria cases and 1,604 community controls) ^12^ for whom data were also available for α-thalassaemia genotypes, directly typed by PCR ^19^. The key characteristics of the samples included in this current study are summarized in Table 1.

### Haplotype structure and LD in the *HBA* region

First, we used statistical phasing to construct haplotype maps for the 400kb region surrounding the -α ^3.7I^ deletion. Briefly, we merged the directly typed α-thalassaemia genotype data with genome-wide SNP data (mapped to GRCh37) in a 10Mb region surrounding the -α ^3.7I^ allele with a view to capturing any potential long-range recombination structure. We phased haplotypes using SHAPEIT2 (see Methods and Extended Data Section 1). We then focused on a 400kb region (chr16:84870-398421) (chr 16:~225818bp [ Figure 1], avoiding the telomere), that included 160kb 5’ and 173kb 3’ flanking the -α ^3.7I ^deletion. This 400kb region contained 178 SNPs (75 SNPs 5’ and 103 SNPs 3’ to α-thalassaemia) that had a minor allele frequency (MAF) of >1% in this dataset (Underlying Data1 Table BB_0-400kb_snp_details and Extended Data). Although statistical phasing is itself based on a model of haplotype copying which could be problematic for complex mutations, we noted that observed haplotypes were supported by the 423 (14%) homozygous carriers in our sample set ( Table 1), suggesting that phasing was generally accurate.

**Figure 7.**
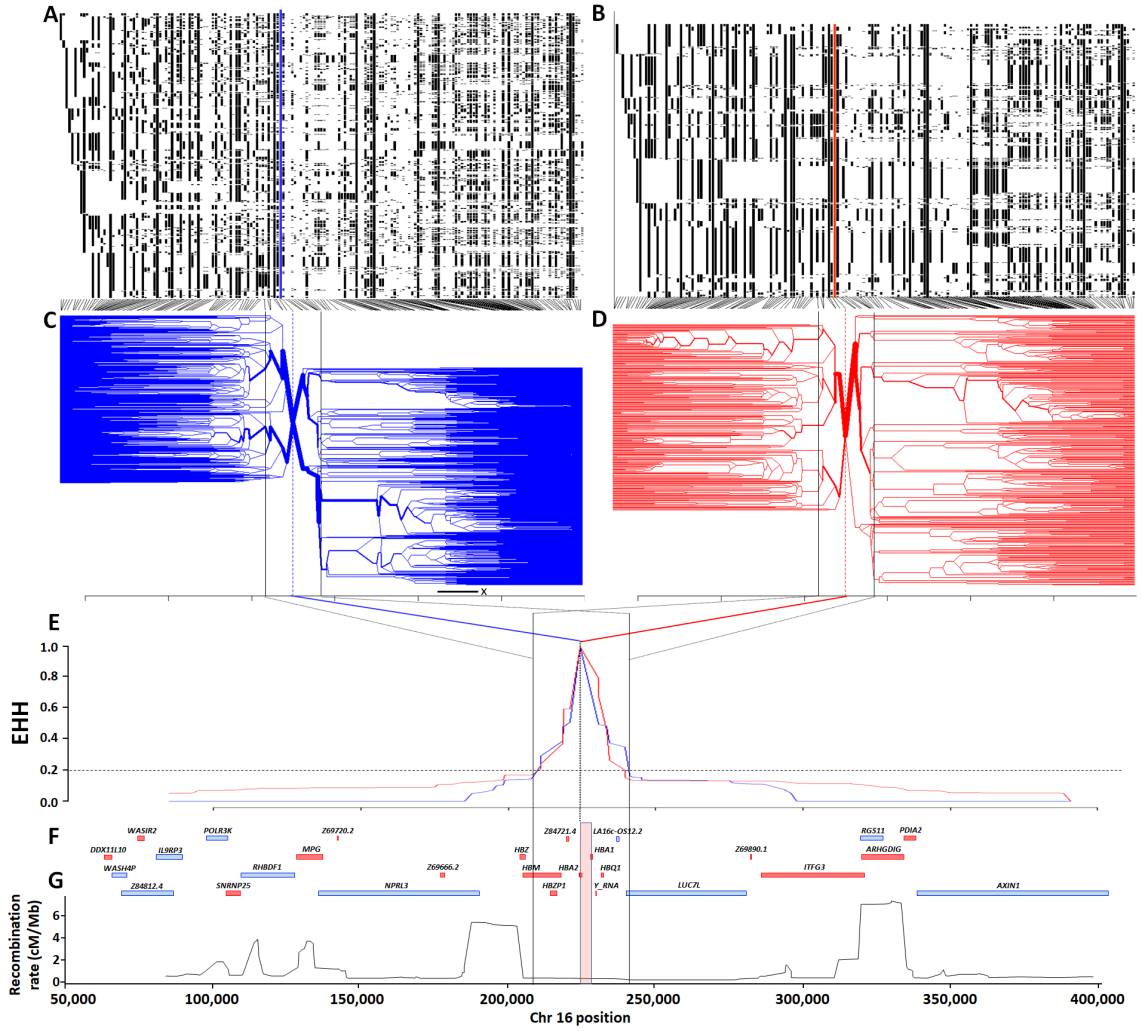
Haplotype, extended haplotype homozygosity (EHH) and bifurcation diagrams for the *HBA* region in Kilifi, Kenya. Panels **A** and **B** show haplotype maps for the -α ^3.7I^-reference and -α ^3.7I^-deletion haplotypes respectively (chromosomes are aligned as rows and SNPs in columns; white = reference and black = alternate), while panels **C** and **D** show corresponding bifurcation diagrams for the -α ^3.7I^-reference and -α ^3.7I^-deletion haplotypes respectively. Panel **E** shows EHH plots for -α ^3.7I^-reference and -α ^3.7I^-deletion haplotypes with the deletion allele as the focal point. Panels **F** and **G** show a gene map, and a recombination map based on African sequence data. The red and blue vertical lines in panels **C** and **D** denote the position of the -α ^3.7I^–INDEL, as does the pink vertical box in panel **G** which is scaled to the width of the deletion.

To inspect haplotype structure, we clustered haplotypes separately into -α ^3.7I^ deletion carriers and non-carriers ( Figure 7A and 7B, respectively). On first inspection the haplotypic structure of chromosomes containing the -α ^3.7I^ deletion appeared more limited than that of ancestral chromosomes, but this was probably attributable to the different haplotype numbers (2,320 versus 3,752 distinct haplotypes, respectively; [ Table 1 and Underlying Data2 Table DD]). Similarly, no obvious differences were seen in the degree of extended haplotype homozygosity (EHH) ^35^ between ancestral and derived haplotypes ( Figure 7C–E and Underlying Data2 Table HH_EHH_values). The EHH peaks of both the derived and ancestral chromosomes showed similar steep decreases to an EHH value of ~0.2, before reaching plateaus on either side of the deletion, each creating a similar region approximately 33kb around the -α ^3.7I^ deletion (~16:207909-241210, Underlying Data2 Table HH_EHH_values and Extended Data Section 2). Fifteen SNPs present within this region (with the exception of the *HBZ* [ζ2] gene), span the α-globin gene cluster ( *HBZP1* [ψζ1] to *HBQ1* [θ1]), which is bounded at the 5' end by a large 5.4 cM/Mb recombination peak ( Figure 7G and Underlying Data2 Table CC_chr16_recombination) in the African recombination map data. However, we saw no such obvious recombination peak at the 3' end, the next large 7.3 cM/Mb peak being situated a further 50kb away from this region ( Figure 7G and Underlying Data2 Table CC_chr16_recombination). This latter peak corresponded with a noticeable change in haplotype bifurcation, suggesting that it might have affected the haplotype structure in both the ancestral and derived haplotypes ( Figure 7C and D).

**Figure 8.**
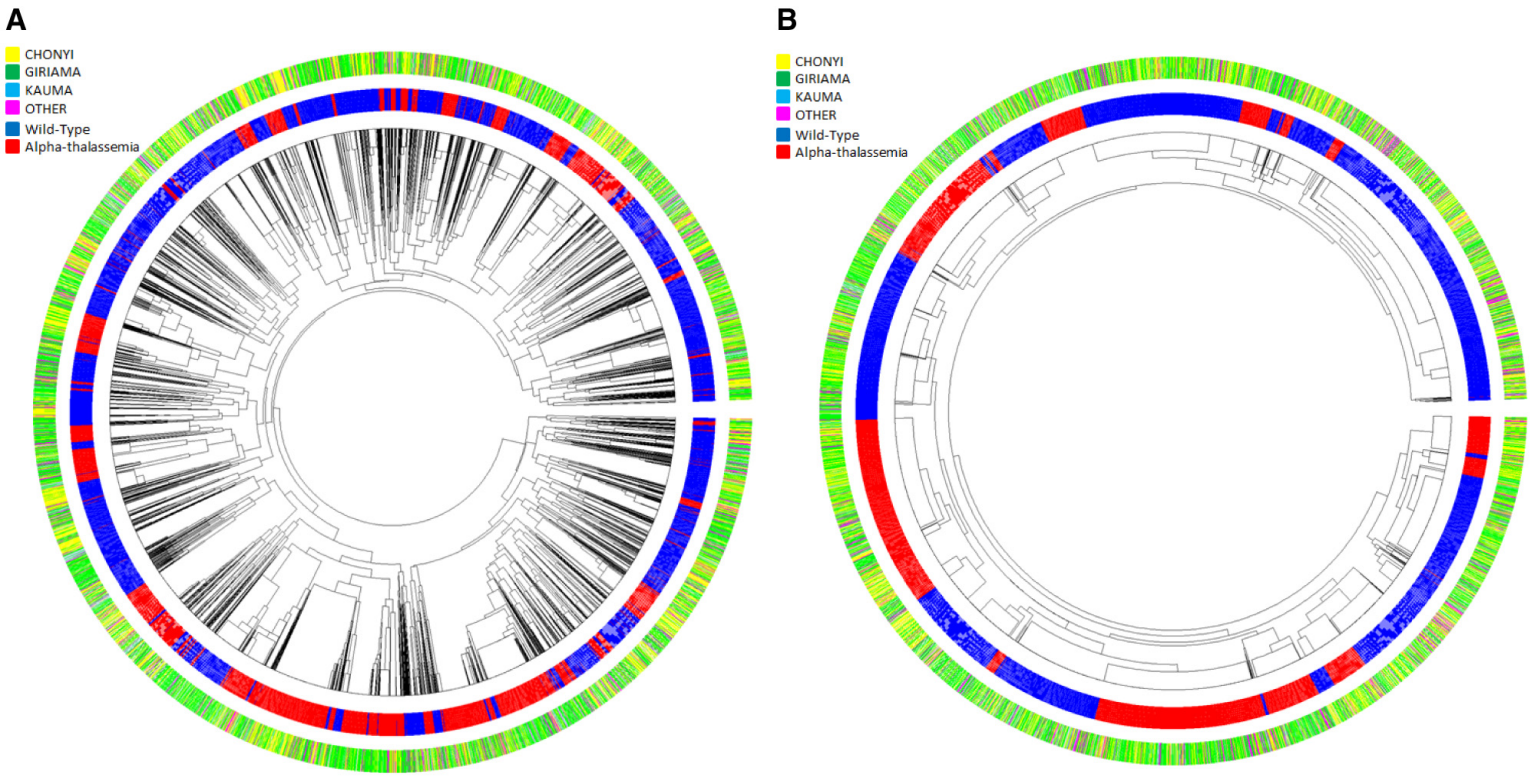
Circular haplotype dendrograms for polymorphisms across the *HBA* region of chromosome 16 for 6072 chromosomes from coastal Kenyan individuals. **A**: Haplotype dendrogram for the full haplotypes of 179 polymorphisms (chr16:84870-398421). **B**: Haplotype dendrogram for the core 33kb region surrounding the -α ^3.7^ deletion (16:207909-241210). Centre shows the dendrogram; middle ring shows individual haplotypes (blue = wt, red = α-thalassaemia); outer ring shows the four major ethnic groupings [Yellow = Chonyi, Green = Giriama, Light Blue = Kauma and Magenta = 'other' groups.

**Figure 9.**
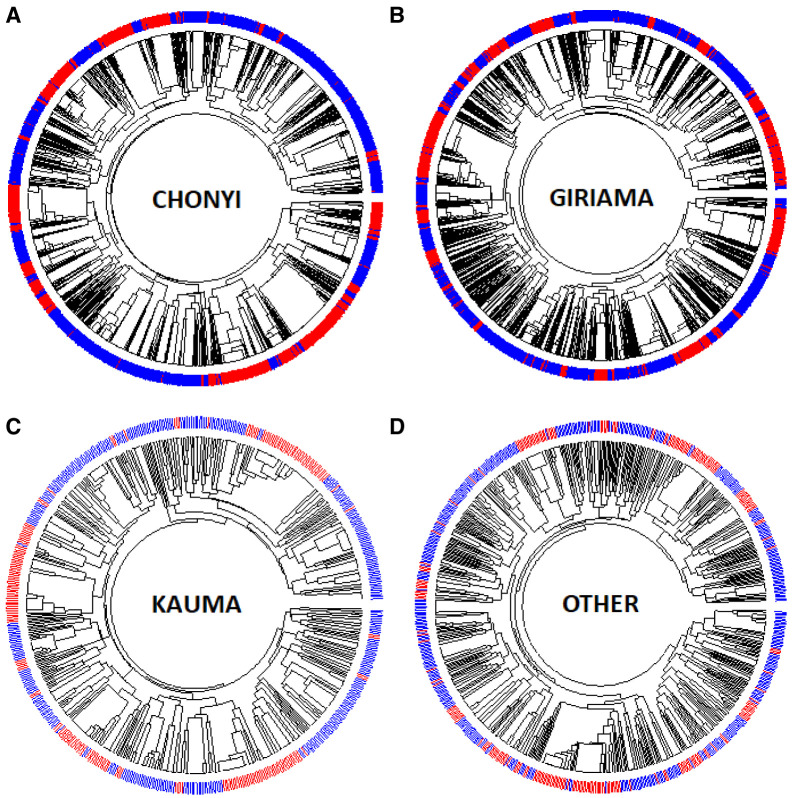
Circular dendrograms of 'core' *HBA* regional haplotypes (Chr16:207,909-241,210 comprising 178 polymorphisms and the α ^-3.7I^ polymorphism) by ethnicity in Kilifi, Kenya. This core region is defined by the EHH signal > 0.2 (Extended Data Figure 2). **A**: Full haplotypes for 178 SNPs and the -α ^3.7I^-thalassaemia locus for the Kauma ethnic group. **B**: Full haplotypes for 178 SNPs and the -α ^3.7I^-thalassaemia locus for the Chonyi ethnic group. **C**: Full haplotypes for 178 SNPs and the -α ^3.7I^-thalassaemia locus for the Giriama ethnic group. **D**: Full haplotypes for 178 SNPs and the -α ^-3.7I^-thalassaemia locus for other ethnic groups having too few haplotypes per group to display individually. -α ^3.7I^-reference (BLUE) and -α ^3.7I^-deletion (RED) haplotypes.

Dendrograms (drawn using the R package Pegas) of haplotypes built across the 400kb region or across the ~33kb region where little recombination was observed ( Figures 8A and 8B, and Extended Data Figures 3–9) showed that haplotypes containing the -α ^3.7I^ deletion were spread throughout the tree (branches marked in red in Figures 8A and 8B, and Underlying Data2 Tables DD_All_Haplotype_frequencies and EE_Core_Haplotype_frequencies). This was not simply explained by variation between ethnic groups ( Figure 9, Extended Data Figures 3–9 and Underlying Data2 Table FF_Ethnic_group_haplotypes), as shown by the track in Figure 8 that shows that the ethnic groups were spread throughout the haplotype tree.

In total, we identified 1,457 haplotypes formed from 179 polymorphisms across the 400kb region, which comprised 1,005 and 452 distinct forms among WT and -α ^3.7I^ individuals, respectively (Underlying Data2 Table DD_All_Haplotype_frequencies). The haplotypes showed similar frequency distributions in WT and -α ^3.7I^ subjects, the maximum frequencies of any single haplotype being 2.5% and 8.3%, respectively. We identified 74 ancestral and 48 derived haplotypes within the 33kb core region, the majority having frequencies of <2.5% (Underlying Data2 Table EE_Core_Haplotype_frequencies). Three haplotypes that were present in both the ancestral and derived groupings had frequencies between 2.5% and 17.5%, while one -α ^3.7I ^haplotype was observed at a frequency of 22.5%. Finally, we calculated diversity scores ^18^ which varied from 0.8-1.0 for all haplotype groups, and Tajima's D’ statistic ^36^ which varied from 1.6-3.8 (Underlying Data2 Table GG_haplotype_metrics), showing that the ancestral and derived haplotypes were similar in structure and diversity across this 400kb region.

### The only genetic form of α-thalassaemia found in the Kilifi study population was -α ^3.7I^

Given the extensive haplotype diversity around the -α ^3.7I ^deletion, we used sequencing to confirm the type of -α ^3.7^ α-thalassaemia deletion that was present within our study population and to investigate the genetic structure of the region in further detail. We used Sanger sequencing of α-thalassaemia PCR reaction products ^19^ from twenty-five -α ^3.7^ and nine WT homozygous individuals. We first compared the sequence across the 3' gene region spanning the four restriction sites between the IVS2 sequence and the 3.7-R PCR primer Figure 2) that is commonly used to determine the type of -α ^3.7^ deletion present ^10^. In all twenty-five -α ^3.7^ homozygous samples, the sequences and paralogous bases for both IVS2 and the four restriction sites ( Table 3 and bases numbered 8–14 in Figure 2) matched the *HBA1* reference sequences (human genome reference *HBA1* sequence [GRCh37] and the sequences from the *HBA1*-specific PCR amplicons from nine Kenyan control individuals). The amplicon region 5' to the IVS2 site spanning the remainder of the *HBA1/2* gene region matched the *HBA2* reference sequences. More specifically, the deletion-amplicon sequences matched the Kenyan *HBA2* sequences; we note that several paralogous base differences in the human reference sequence ( Table 3 and bases numbered 4–6 in Figure 2) were identical and invariant between *HBA1* and *HBA2* in the nine Kenyan control samples. From these data we concluded that the -α ^3.7^ deletion in these Kenyan samples was Type I ^10^, but there was also evidence from variation observed for paralogous base 7 that there may be at least two breakpoint sub-types (Further details are given in Extended Data Section 3).

### A strong signal of association was seen between the -α ^3.7I^ allele and severe malaria, but no signals were seen at any of the SNPs in the surrounding region

**Figure 10.**
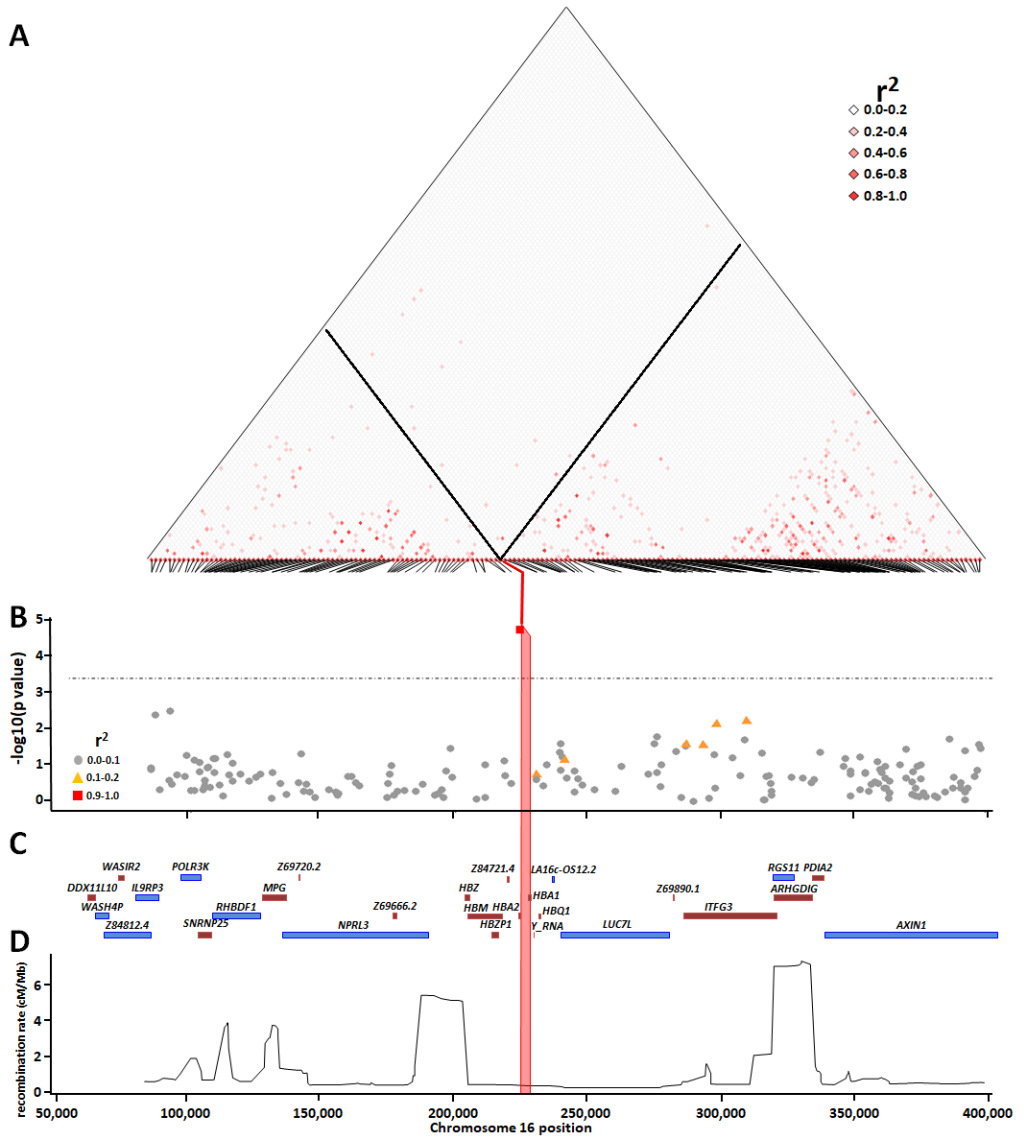
The association between the -α ^3.7I^ deletion and SNPs within the surrounding region with severe malaria. Panel **A** shows pairwise squared correlations (r ^2^) between genotypes at regional variants. Black lines denote the values for -α ^3.7I^ deletion for SNPs within the region. The angled lines at the base of the linkage disequilibrium map identify each SNP and where it aligns to the chromosome. Panel **B** shows a Manhattan plot illustrating the p-values for the associations between individual SNPs within the *HBA* region and severe falciparum malaria; the horizontal dotted line shows the Bonferroni-correction significance threshold (p<0.0003); r ^2^ shows the correlation between α-thalassaemia and the other SNPs. Panels **C** and **D** show the gene map and recombination map, respectively. The pink vertical box in panels **B**, **C** and **D** shows the location of -α ^3.7I^ deletion.

We were interested to know whether the signal of association between the -α ^3.7I^ allele and severe malaria could be replicated by use of any of the SNPs within the surrounding genetic region. Consistent with our previous analyses of the full data set from this case-control study ^12^, we found strong evidence for associations between the -α ^3.7I^ deletion, typed directly by PCR, and all forms of severe *P. falciparum* malaria in this current sub-analysis. The adjusted odds ratio (aOR) for severe malaria overall was 0.78 (95% CI 0.70-0.87; p=11×10 ^-6^) ( Figure 10B and Underlying Data2 Tables II _assoc_results_unadjusted and JJ _assoc_results_adjusted_hbs), while on genotypic analysis, heterozygosity (-α/αα) and homozygosity (-α/-α) were associated with aORs of 0.79 (95% CI 0.67-0.93, P=0.005) and 0.59 (95% CI 0.47-0.76, P=2.43×10 ^-5^), respectively. Nevertheless, based on a Bonferroni-corrected significance threshold of P<0.0003, we found no significant associations at any of the 178 SNPs in the surrounding region ( Figure 10B). Although two SNPs telomeric to the -α ^3.7I^ deletion (rs62031426 [kgp4990237] and rs41340949 [kgp1941708]), were marginally associated (P=0.002 and 0.003, respectively), both were in low LD with the -α ^3.7I^ deletion (r ^2^ < 0.01; Figure 10B and Underlying Data2 Table LL_pairwise_R2) and the weak associations at these SNPs were therefore unlikely to have been reflective of α-thalassaemia. As noted by others ^2^, the absence of associations at SNPs within this region is most likely explained by low LD ( Figure 10A and Underlying Data2 Tables KK_pairwise_R, LL_pairwise_R2 and MM_pairwise_Dprime). The maximum r ^2^ values between -α ^3.7I^ and any of the surrounding 178 SNPs being 0.081 (rs170058) and 0.16 (kgp4999044 [rs11863726]) in the 3’ and 5’ regions, respectively (Underlying Data2 Table LL_pairwise_R2).

### Predicting genotypes using haplotype structure with Impute 2

Given the availability within our dataset of both phased data, α-thalassaemia genotypes and haplotypes, we were able to investigate the prediction of α-thalassaemia genotypes using IMPUTE2 software ^33^. This method requires a set of reference haplotypes that have been genotyped for α-thalassaemia. To evaluate imputation, we conducted a cross-validation experiment in which we repeatedly selected 100 individuals (200 chromosomes) at random from our total sample set to form a reference panel. We then imputed the α-thalassaemia genotypes for the remaining samples before calculating concordance with the directly typed results. We repeated this exercise 1,000 times with a view to estimating the imputation performance that would be expected if a reference panel for this population were available ( Table 4, Extended Data Section 6, and Underlying Data2 Table AD_Impute2_overall_summary).

Overall, depending on the genotype and threshold, we were able to predict the true genotypes with sensitivity of 62–77%, specificity of 88–98% and positive prediction value (PPV) of 83–94% ( Table 4 and Table 5) (further details in Extended Data Section 6). The correlation of the imputed genotype calls was 0.82 ± 0.03 and 0.84 ± 0.03 (mean ± SD) for the 70% and 90% genotype-calling threshold, respectively (using called samples only; 2,639 ± 120 and 2,193 ± 212 genotype calls, respectively [mean ± SD]) (Extended Data Figure 34 and Underlying Data2 Table AM_imputation_correlations). Together this correlation and reduced sample size will have the effect of reducing association power.

**Table 4.** Metrics from Imputation bootstrapping (1,000 runs).

metric	Number of correct genotypes	Total number of genotypes	No-call number	Correct Proportion of genotypes	Adjusted- correct proportion of genotypes	Missing proportion of genotypes	Wild-Type Homozygote				-α ^-3.7I^ Heterozygote				-α ^-3.7I^ Homozygote			
							sensitivity	specificity	accuracy	PPV	sensitivity	specificity	accuracy	PPV	sensitivity	specificity	accuracy	PPV
**70% threshold**																		
**Minimum**	1645	2936	54	0.560	0.746	0.018	0.582	0.826	0.796	0.582	0.486	0.787	0.710	0.486	0.256	0.910	0.889	0.256
**1 ^st^ Qu**	2173	2936	206.8	0.740	0.836	0.070	0.736	0.924	0.859	0.736	0.745	0.862	0.807	0.745	0.696	0.962	0.929	0.696
**Median**	2259	2936	274.5	0.769	0.854	0.093	0.770	0.938	0.872	0.770	0.776	0.877	0.827	0.776	0.755	0.970	0.938	0.755
**Mean**	2248	2936	296.2	0.766	0.852	0.101	0.765	0.934	0.871	0.765	0.772	0.875	0.825	0.772	0.747	0.968	0.937	0.747
**SD**	123.92		120.15	0.040	0.025	0.040	0.050	0.020	0.020	0.050	0.050	0.020	0.030	0.050	0.090	0.010	0.010	0.090
**3 ^rd^ Qu**	2334	2936	361.2	0.795	0.869	0.123	0.800	0.948	0.883	0.800	0.807	0.891	0.844	0.807	0.809	0.976	0.946	0.809
**Maximum**	2555	2936	1027	0.870	0.911	0.350	0.895	0.976	0.922	0.895	0.870	0.933	0.891	0.870	0.912	0.992	0.965	0.912
**90% threshold**																		
**Minimum**	1170	2936	215	0.399	0.771	0.073	0.453	0.851	0.754	0.453	0.302	0.829	0.639	0.302	0.134	0.932	0.876	0.134
**1st Qu**	1791	2936	589.8	0.610	0.861	0.201	0.640	0.940	0.833	0.640	0.597	0.902	0.760	0.597	0.517	0.976	0.918	0.517
**Median**	1938	2936	716	0.660	0.878	0.244	0.681	0.953	0.849	0.681	0.656	0.916	0.789	0.656	0.621	0.981	0.930	0.621
**Mean**	1919	2936	742.8	0.654	0.875	0.253	0.678	0.949	0.847	0.678	0.647	0.915	0.785	0.647	0.612	0.980	0.929	0.612
**SD**	194.51		212.13	0.066	0.025	0.072	0.062	0.018	0.022	0.062	0.081	0.020	0.038	0.081	0.137	0.008	0.017	0.137
**3rd Qu**	2059	2936	880	0.701	0893	0.300	0.721	0.961	0.862	0.721	0.706	0.929	0.813	0.706	0.717	0.985	0.941	0.717
**Maximum**	2436	2936	1544	0.830	0.925	0.526	0.840	0.981	0.901	0.840	0.830	0.960	0.873	0.830	0.887	0.997	0.965	0.887

Adjusted-correct proportion was calculated for each run by adjusting the total number of genotypes by the number of no-called genotypes (Underlying Data2 Table AD_Impute2_overall_summary).

**Table 5.** Summary of the performance of methods using haplotypes or intensities to infer the -α ^-3.7I^ genotypes from features/SNPs within the -α ^-3.7I^ deletion region.

Methods	Principle	Directly typed (N)	Sensitivity ^***^			Specificity ^***^			Positive Predicative Value ^***^			Overall Association (OR [95%CI] p-value)	Model	Genotypic Association (OR) adjusted		
			Norm	Het	Homo	Norm	Het	Homo	Norm	Het	Homo			Norm (ref)	Het	Homo
Direct genotyping	PCR genotyping	3036	NA			NA			NA			0.78 (0.70-0.87) (p=1.1x10 ^-5^)	Additive	1	0.79 [0.67-0.93] (p=0.005)	0.60 [0.47-0.76] (p=2.43x10 ^-5^)
Intensity distributions (4 features) ^*^	Intensity thresholds (AF3;XX)	0	24.1	83.1	93.1	91.3	44.7	95.8	81.4	61.8	78.2	0.76 [0.64-0.9] (p=0.0013)	Homozygous or Heterozygous	1	0.74 [0.62-0.88] (p=7.2x10 ^-4^)	0.83 [0.66-1.04] (p=1.11x10 ^-11^)
			to	to	to	to	to	to	to	to	to	to			to	to
			63.2	94.8	95.2	98.8	72.9	98.6	92.6	74.4	91.8	1.74 [1.42-2.13] (p=5.8x10 ^-8^)			1.87 [1.43-2.44] (p=4x10 ^-6^)	1.49 [1.06-1.98] (p=2.1x10 ^-9^)
Hierarchical clustering (5 features)	Hierachical clustering (AF3;TT)	0	56	74	96	95	71	86	80	73	88	1.43 [1.28-1.69] (p=1.3x10 ^-10^)	Additive	1	1.12 [0.91-1.37] (p=0.05)	2.02 [1.6-2.55] (2.2x10 ^-9^)
Hierarchical clustering (4 features)	Hierarchical clustering (AF3;TT)	0	41	74	96	93	61	87	73	67	88	1.86 [1.56-2.22] (p=4.98x10 ^-12^)	Recessive	1	1.24 [1.02-1.49] (p=0.02)	2.02 [1.62-2.51] (p=2.5x10 ^-10^)
MRM ^*^	Regression (AF3;VV)	100	58	75	79.8	83.1	53.4	96.6	70.1	61.7	71.6	0.79 [0.68-0.92] (p=0.0025)	Heterozygous or Dominant	1	0.8 [0.67-0.95] (p=0.01)	0.81 [0.65-1.02] (p=0.08)
			to	to	to	to	to	to	to	To	to	to			To	to
			73	86	85	89.6	75.3	98.6	79.2	76.7	90.1	1.67 [1.45-1.94] (p=3.7x10 ^-12^)			1.63 [1.37-1.95] (p=3.5x10 ^-8^)	2.61 [2.05-3.33] (p=6.8x10 ^-15^)
CART ^*^	Hierarchical clustering and regression (AF3;UU)	100	68.1	45.5	68.6	76.7	61.3	96.7	62.4	61.4	67.6	0.799 [0.69-0.93] (p=0.004)	Heterozygous, or Dominant	1	0.79 [0.67-0.93] (p=0.01)	0.81 [0.64-1.02] (p=0.08)
			to	to	to	to	to	to	To	To	to	to			To	to
			78.8	86.7	97.2	97.7	81.6	99.1	84.4	81.4	92.9	1.70 [1.47-1.97] (p=1.19x10 ^-12^)			1.72 [1.44-2.04] (p=9.8x10 ^-9^)	1.9 [0.85-1.39] (0.47)
IMPUTE2 ^**^ 70% threshold (means ± SD)	Imputation (1000 runs) (AF3;AG/AI)	100	77	77	75	93	88	97	87	83	94	0.74 ± 0.07 (p =1.71x10 ^-3^ ± 3x10 ^-3^)	Additive, dominant or recessive		0.79 ± 0.04 (p=0.02 ± 0.04)	0.61 ± 0.04 (p=0.009 ± 0.003)
			±	±	±	±	±	±	±	±	±			1		
			5	5	9	2	2	1	2	3	1					
IMPUTE2 ^**^ 90% threshold (means ± SD)	Imputation (1000 runs) (AF3;AH/AJ)	100	68	66	62	95	92	98	85	79	93	0.76 ± 0.08 (p = 0.005 ± 0.010)	Additive, dominant or recessive		0.77 ± 0.05 (p=0.03 ± 0.06)	0.61 ± 0.05 (p=0.004 ± 0.013)
			±	±	±	±	±	±	±	±	±			1		
			6	8	14	2	2	1	2	4	2					

Further details of methods and results can be found in the Extended Data. * Where several SNPs (as indicated) were tested, then the ranges of results are shown. ** Data are mean ± SD for 1,000 individual runs of IMPUTE2 (70% and 90% refer to the threshold used to assign a genotype). *** Data are shown as percentages. MRM, Multiple-Regression Model; CART, Classification and Regression Trees.

### α-thalassaemia genotype was correlated with intensity signals for SNPs within the -α ^3.7I^ deletion

**Figure 11.**
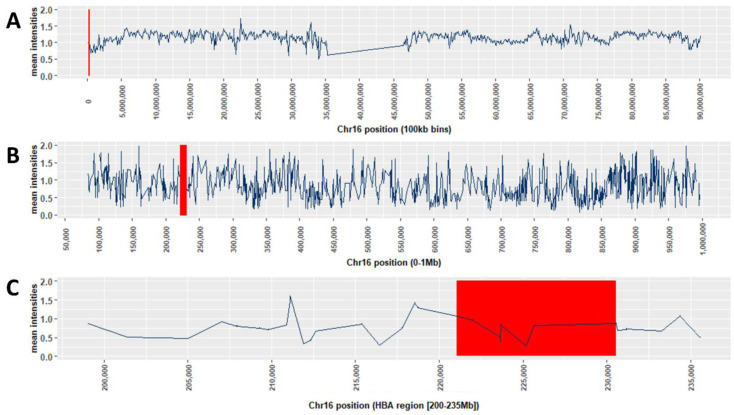
Mean of the Sum(X + Y) intensities for 3036 samples for SNP features on chromosome 16. **A**: Mean channel intensities (X + Y) for 3036 samples used in the study, averaged over 100kb bins for chromosome16 **B**: Mean channel intensities (X + Y) for 3036 samples for individual SNPs in the 5’ 0-1Mb telomereic region of chromosome 16 **C**: Mean channel intensities (X + Y) for 3036 samples for individual SNPs in the *HBA* gene region of chromosome 16 The red rectangle in each plot shows the location of the -a ^3.7^ deletion.

Given that we were unable to predict α-thalassaemia genotypes with sufficient reliability through the use of LD or imputation methods, we next investigated the potential for an alternative approach – the use of intensity data at SNPs that lie within the deleted region (Underlying Data2 Table OO_intensity_summary). For the purpose of these analyses, we therefore summed the chip channel intensities (X + Y) to produce a single total intensity value for each individual across each SNP across chromosome 16. We first plotted these intensities averaged over 100kb bins for the whole chromosome ( Figure 11A) showing that there was an end-of-chromosome reduction in intensities (from ~ 1.25 to ~ 0.75). At the individual SNP level the mean intensity for the 0-1Mb 5' telomere region where the *HBA* region lies ( Figure 11B) was ~0.75-1.

**Figure 12.**
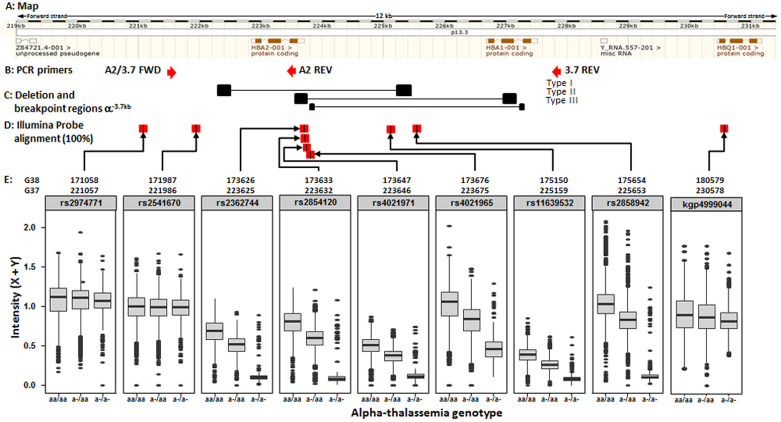
Intensity plots of the Illumina 2.5M chip features across the -α ^3.7^kb deletion. **A**: Map of the Human *HBA2* and *HBA1* region on chromosome 16 ( http://www.ensembl.org/index.html), with respect to the forward strand (GRCh37 coordinates). **B**: Primers used for the α ^WT^ and -α ^3.7kb^ deletion are shown by red arrows below the gene map (A2/3.7 FWD, A2 REV, 3.7 REV). **C**: The -α ^3.7^ deletions are shown below the gene map and are highlighted according to Hill *et al.* ^37^ and data from this study (see main text). **D**: Feature probes with 100% match to the reference sequence are shown below the gene map by red boxes with a black vertical line. **E**: Coordinates for each feature are given for both GRCh37 and GRCH38 coordinates. **F**: Plots of the sum of the X + Y channel intensities (Y-axis) from the chip and PCR-typed genotype (X-axis) for each SNP (αα/αα WT; -α/αα -3.7kb HET, -α/-α -3.7kb HOM).

The *HBA* region lies between 200kb and 235kb from the 5' telomeric end where the mean intensities were ~ 0.75 ( Figure 11B and C, and Extended Data Figure 12-14). While this may be sufficient to have some impact on the predicative performance of the data, we hypothesized that intensity signals for such SNPs within the deletion region would still be reduced in α-thalassaemic subjects and that reductions would be dose-dependent in such a way that they would be greatest in homozygotes. We identified six features (rs2362744, rs2854120, rs4021971, rs4021965, rs11639532 and rs2858942) on the Illumina HumanOmni2.5-4 chip that lay within the -α ^3.7I^ deletion, along with three flanking SNPs that served as controls ( Figure 12 and Underlying Data2 Table OO_intensity_summary). The Sum(X+Y) data for each individual and each of the 6 SNPs were then plotted as means with 25 ^th^ and 75 ^th^ percentiles and outliers on the y-axis with stratification by α-thalassaemia genotype on the x-axis ( Figure 12, Extended Data Figures 16 and as scatter plots Extended Data Figure 17). As anticipated, we saw significant step-wise reductions in intensities by α-thalassaemia genotype for all six features within the deletion (Kruskall-Wallis and Dunn's test for multiple comparisons ^21^; Extended Data Section 4, Underlying Data2 Tables PP_intensity_comparisons and QQ_intensity_summary), leading us to investigate a range of potential methods for predicting α-thalassaemia genotypes from such data.

### Signal intensities alone were not enough to predict α-thalassaemia genotype with any accuracy

Based on the assumption that most investigators would not have access to direct genotyping, we first created histogram and density plots of the intensity data from the nine SNPs described above, without reference to data on α-thalassaemia genotype ( Figure 4). From these data we identified troughs or shoulders to separate the different genotype classes; SNPs rs4021965 and rs2858942 did not show a trough or shoulder to help identify heterozygotes from WT normal individuals, although they did show some discrimination of the homozygotes. But four SNPs (rs2362744, rs2854120, rs4021971, rs11639532) did show possible breaks between each genotype class ( Figure 4 and Extended Data Table 3 and Underlying Data2 Table RR_intensity_cutoffs) and we focused on these to infer α-thalassaemia genotypes from these groupings (Extended Data Figure 21 and Underlying Data2 Table RR_intensity_cutoffs). The overall ROC curves (Extended Data Figure 22) suggested good prediction of -α ^3.7I^ homozygous individuals by this approach when using data from any of the four aforementioned SNPs (78–92% PPV, 93–95% sensitivity and 96–99% specificity; Table 5 and Extended Data Figure 22, Underlying Data2 Table SS_crude_genotype_assignment). However, such data were not useful in distinguishing heterozygotes from WT normal individuals, for which the PPVs were 62 and 93%; sensitivity between 24 and 95%; and specificity between 45 and 99% ( Table 5 and Underlying Data2 Table SS_crude_genotype_assignment). In an extension to this method we used hierarchical clustering for the six SNPs described above in the deletion region described above ( Figure 5 and Extended Data Figure 23 and Underlying Data2 Table TT_del_hier_cluster_assignment) and due to differences in intensity profiles we also investigated a reduced set of four or five SNPs ( Figure 5 and Extended Data Section 4c). Genotypes were assigned based on the three primary tree branches in each model but did not improve performance for inferring α-thalassaemia genotypes (80%, 73% and 88% PPV for αα/αα – α/αα and -α/-α respectively; Table 5 and Underlying Data2 Table TT_del_hier_cluster_assignment).

### α-thalassaemia genotype-prediction was not improved by using modelling approaches to interpret SNP intensity data

Given the poor predictive value of the raw intensity data as is, we investigated the potential utility of an alternative approach using models trained with a subset of directly genotyped samples. We tested two methods - a multinomial regression model (MRM) and a Classification and Regression Tree (CART) model - each of which used both the six SNPs within the deletion individually and all six SNPs combined. For both methods we used a bootstrap approach to test each sample selection 1,000 times using training sets of between 10 and 500 individuals as described in detail in Extended Data Section 5. We found that the minimum training sets that were required to reach a plateau of predicative power of the inferred versus real genotypes included just 100 samples, although the CART method appeared to produce a less stable prediction (Extended Data Figure 27). Depending on which SNP was included, α-thalassaemia homozygotes were predicted with sensitivities and specificities of 58–97% and 97–99%, respectively ( Table 6, and Extended Data Figure 27). Predictions from models based on all six SNPs combined did not improve performance.

**Table 6.** Performance of the MRM and CART models for the prediction of α-thalassaemia genotype.

Method	SNPID	Genotypes	Sensitivity %	Specificity %	PPV %	NPV%
			(95%CI)	(95% CI)	(95% CI)	(95% CI)
**MRM**	rs2362744	αα/αα	64.8 (62.0-67.7)	87.6 (86.1-89.1)	75.9 (73.4-78.1)	80.7 (79.3-81.9)
		–α/αα	82.5 (80.5-84.5)	71.5 (69.2-73.8)	73.1 (71.5-74.7)	81.4 (79.4-83.1)
		–α/–α	84.8 (81.0-88.2)	98.5 (97.9-98.9)	90.1 (86.9-92.5)	97.5 (96.9-98.0)
	rs2854120	αα/αα	66.3 (63.5-69.1)	89.6 (88.1-91.0)	79.2 (76.8-81.5)	81.6 (80.3-82.9)
		–α/αα	85.7 (83.8-87.5)	75.6 (73.4-77.8)	76.7 (75.1-78.3)	85.0 (83.2-86.5)
		–α/–α	95.1 (92.7-97.0)	98.6 (98.2-99.1)	92.3 (89.5-94.4)	99.2 (98.8-99.5)
	rs4021971	αα/αα	70.2 (67.4-72.9)	87.3 (85.8-88.9)	76.9 (74.6-79.1)	83.1 (81.7-84.3)
		–α/αα	82.2 (80.2-84.2)	74.7 (72.5-77.0)	75.4 (73.7-77.0)	81.8 (80.0-83.5)
		–α/–α	81.4 (77.4-85.1)	98.3 (97.8-98.8)	89.0 (85.6-91.6)	97.0 (96.3-97.5)
	rs4021965	αα/αα	57.5 (54.5-60.5)	86.9 (85.4-88.5)	72.6 (69.9-75.0)	77.4 (76.1-78.6)
		–α/αα	80.0 (77.9-82.1)	53.4 (50.9-56.0)	61.7 (60.3-63.2)	74.1 (71.9-76.2)
		–α/–α	58.2 (53.5-63.1)	97.5 (96.8-98.1)	71.6 (65.8-76.8)	90.5 (89.9-91.2)
	rs11639532	αα/αα	72.6 (69.9-75.2)	88.6 (87.1-90.0)	79.2 (77.0-81.3)	84.4 (83.1-85.6)
		–α/αα	82.9 (80.8-84.8)	74.4 (72.2-76.6)	75.3 (73.6-76.9)	82.3 (80.5-83.9)
		–α/–α	85.9 (71.6-89.0)	98.1 (97.5-98.6)	86.8 (83.2-89.8)	96.1 (95.4-96.7)
	rs2858942	αα/αα	64.7 (61.8-67.5)	88.2 (86.7-89.7)	76.7 (74.2-79.0)	80.7 (79.4-81.9)
		–α/αα	80.0 (77.9-82.1)	53.4 (50.9-56.0)	61.7 (60.3-63.2)	74.1 (71.9-76.2)
		–α/–α	81.0 (53.5-83.1)	97.5 (96.8-98.1)	71.6 (65.8-76.8)	90.5 (89.9-91.2)
	Combined	αα/αα	66.0 (63.2-68.9)	83.1 (81.4-84.9)	70.1 (67.8-72.4)	80.4 (79.0-81.7)
		–α/αα	75.0 (72.7-77.3)	72.3 (70.0-74.6)	71.8 (70.0-73.5)	75.5 (73.7-77.3)
		–α/–α	79.8 (75.6-83.6)	96.6 (95.9-97.3)	79.8 (76.1-83.1)	96.7 (96.0-97.2)
**CART**	rs2362744	αα/αα	68.1 (53.8-83.7)	97.7 (77.2-98.9)	62.4 (59.8-64.7)	75.9 (74.4-77.1)
		–α/αα	72.5 (70.2-74.9)	68.4 (66.1-70.8)	68.7 (66.8-70.3)	72.6 (70.6-74.3)
		–α/–α	97.2 (88.9-97.4)	99.1 (98.3-99.2)	92.7 (90.0-94.7)	98.8 (98.2-99.1)
	rs2854120	αα/αα	76.5 (59.7-77.5)	87.6 (85.0-88.2)	73.9 (71.3-76.1)	79.6 (78.2-80.7)
		–α/αα	85.7 (79.7-83.8)	72.9 (70.6-75.2)	74.2 (72.3-75.6)	81.0 (79.2-82.7)
		–α/–α	97.1 (92.6-97.6)	98.5 (98.0-99.0)	91.7 (88.7-93.8)	99.2 (98.8-99.5)
	rs4021971	αα/αα	78.8 (61.8-80.6)	92.7 (90.4-94.0)	82.6 (80.1-84.7)	81.4 (80.0-82.5)
		–α/αα	86.7 (84.9-88.4)	76.6 (72.6-77.8)	76.3 (74.6-77.8)	85.7 (83.9-87.2)
		–α/–α	96.5 (92.6-97.0)	97.5 (96.9-98.1)	86.3 (83.2-89.0)	99.3 (98.8-99.5)
	rs4021965	αα/αα	69.0 (61.4-69.7)	76.7 (74.7-78.7)	62.4 (60.1-64.5)	78.4 (76.7-79.6)
		–α/αα	45.5 (36.7-56.4)	61.3 (57.8-63.8)	61.4 (59.5-63.0)	65.9 (63.8-67.6)
		–α/–α	68.6 (64.1-69.1)	96.7 (96.0-97.4)	67.6 (62.1-72.6)	91.0 (90.3-91.7)
	rs11639532	αα/αα	75.0 (72.3-77.5)	91.7 (90.4-92.9)	84.4 (82.3-86.4)	86.0 (84.7-87.2)
		–α/αα	85.2 (83.3-87.1)	81.6 (79.6-83.5)	81.4 (79.7-83.0)	85.6 (83.8-87.0)
		–α/–α	97.2 (90.3-98.4)	96.7 (95.9-97.4)	82.2 (78.9-85.1)	98.9 (98.4-99.2)
	rs2858942	αα/αα	60.8 (56.9-62.7)	89.6 (88.2-91.0)	77.7 (75.0-80.0)	78.8 (77.6-80.0)
		–α/αα	82.3 (80.3-84.3)	69.3 (66.9-71.6)	71.8 (70.0-73.2)	80.7 (78.8-82.4)
		–α/–α	95.7 (87.6-96.4)	96.9 (96.2-97.6)	82.9 (79.5-85.8)	98.5 (97.9-98.9)
	Combined	αα/αα	73.2 (70.6-75.9)	80.1 (78.1-81.8)	68.7 (66.6-70.8)	83.3 (81.9-84.7)
		–α/αα	73.6 (71.2-75.9)	80.3 (78.1-82.1)	77.7 (75.9-79.5)	76.3 (74.7-77.9)
		–α/–α	93.2 (90.6-95.6)	98.2 (98.3-99.2)	92.9 (90.0-94.8)	98.9 (98.5-99.3)

Metrics were calculated for α-thalassaemia genotypes inferred from MRM and CART models created using 100 randomly selected individuals as a 'training' set, and repeated 1000 times. PPV = Positive predictive value; NPV= negative predictive value; MRM, Multiple-Regression Model; CART, Classification and Regression Trees.

### We found no consistent concordance between association signals derived from direct and predicted α-thalassaemia genotypes

**Figure 13.**
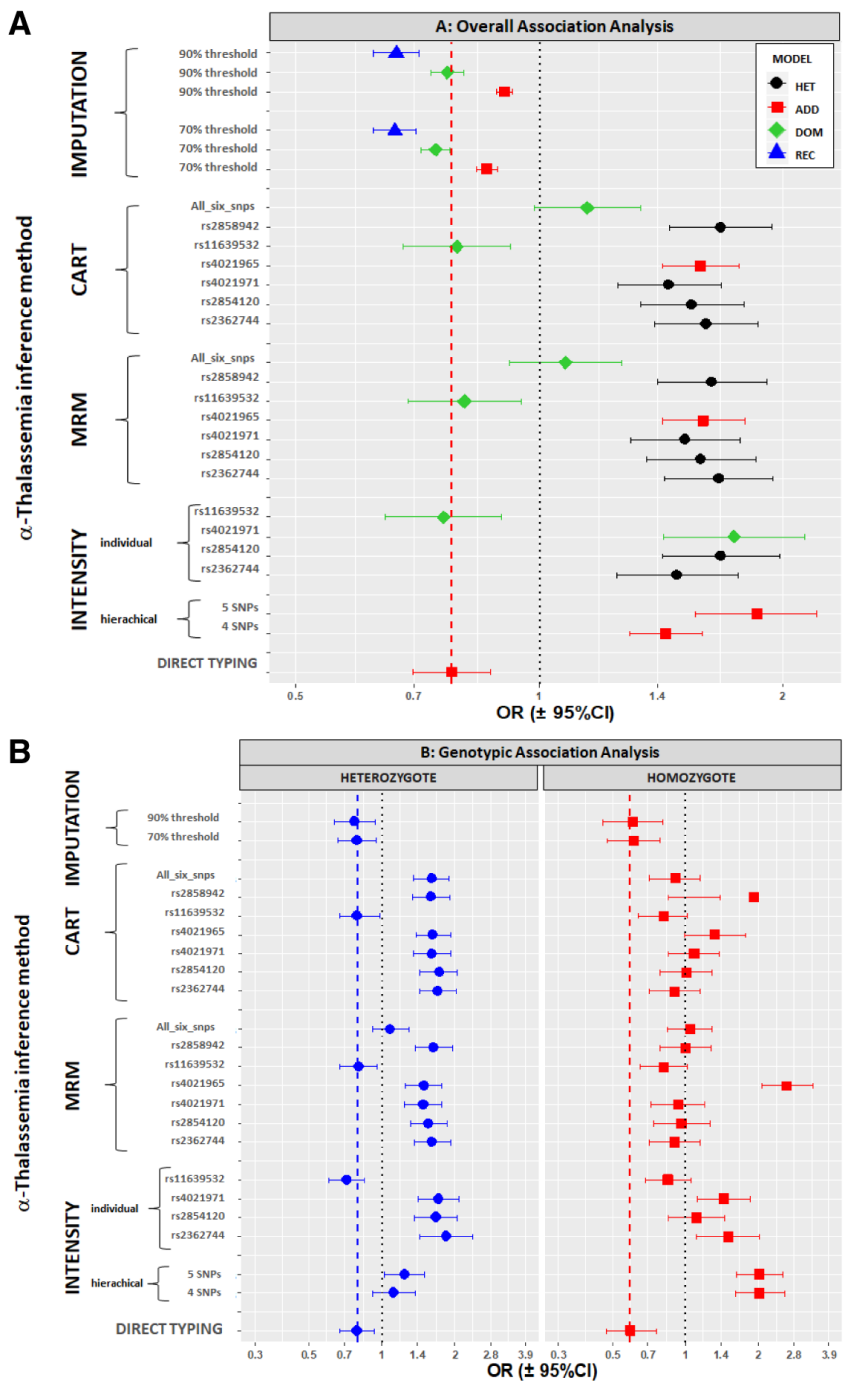
Association of α-thalassaemia with severe malaria by an overall test ( **A**) and by genotypic tests ( **B**). We used various methods to infer α-thalassaemia genotypes and then tested for association with severe malaria as detailed in the y-axis labels (see also main text). For the imputation results, these are mean results across the 1000 runs (see Methods); overall association results are split by the best association model results from each run while genotypic results are for all runs (Extended Data Section 6). The black dashed vertical line shows the no-effect position, while the red or blue vertical dashed lines show the direct typing effects.

Finally, although the models above did not predict the true α-thalassaemia genotypes perfectly, we were interested to see whether they might still provide sufficient information to identify the malaria-protective associations that we saw by direct α-thalassaemia genotyping ( Figure 10, summarised in Figure 13 and Table 5; and Underlying Data2 Tables JJ_assoc_results_adjusted_hbs, UU_crude_intensity_assoc, VV_del_hier_clustering_assoc, WW_MRM_association_results, XX_CART_association_results, AJ_imp_overall_assoc_70, AK_imp_overall_assoc_90, and AL_imp_overall_Pvals, Extended Data Figures 35, 36, Extended Data Tables 4, 5, 7, 8). Although we identified many significant associations across the predictive models for a number of SNPs, we saw no consistent pattern for either the best inheritance models or the direction of association, both of which were often at odds with those of the true associations ( Figure 13 and Table 5). We did note, however, that for genotypic associations, rs11639532 (located in the intra-genic region between *HBA2* and *HBA1*) did give similar results to the directly typed data when using crude intensity cut-offs or MRM or CART models. Similarly, IMPUTE 2 performed reasonably well for genotypic association testing.

## Discussion

In common with many parts of sub-Saharan Africa, the -α ^3.7I^ deletion is present at a high frequency in Kilifi, Kenya, where it not only protects against falciparum malaria, but also affects the risk of other childhood diseases ^12, 17, 38^. Furthermore, in previous studies, we have shown that with regard to its effects on malaria susceptibility, the -α ^3.7I^ deletion can also interact with mutations in unrelated genes ^39– 43^. Taken together, these observations suggest that α-thalassaemia is an important confounder that should be considered when interpreting disease-association studies in Africa. Despite this however, few studies correct for α-thalassaemia because of the current need for direct genotyping. Because an increasing number of GWAS studies are now being conducted in Africa, our main purpose in conducting this study was to investigate whether this might be achievable by the use of indirect GWAS-based data.

In the current study, we confirm the high frequency of α-thalassaemia in the Kilifi population and show that the -α ^3.7I^ deletion is the only mutation responsible according to the current classification ^10^. This was borne out using 25 samples from subjects with the homozygous deletion that we subjected to Sanger sequencing. While all showed the same sequence that was concordant with having the -α ^3.7I^ deletion type (Type 1 ^10^), we found evidence that this Type I form had been created by at least two separate breakpoint events, suggesting that the current classification warrants expanding. Our first aim was then to investigate why we had failed to identify the -α ^3.7I^ signal in our previous GWAS studies of severe malaria ^3, 15^. Focusing on a 400kb region spanning the entire α-globin region, we found that LD, both overall and specifically with the -α ^3.7I^ locus, was very low. This alone provided a good explanation that was further supported by our observations regarding the haplotype structure across the region. We found that the -α ^3.7I ^deletion was distributed throughout the haplotype tree over this entire region, while EHH analysis showed that haplotype structure decayed quickly over ^~^16kb both 5’ and 3’ of the -α ^3.7I^ locus and that this corresponded closely with the span of the α-globin genes ( *HBZ* to *HBQ1*) and a large recombination peak centred on *HBZ*. Similarly, diversity measures were high, and equivalent in both the -α ^3.7I^-containing and reference alleles. Our sequencing data, which suggested that there are at least two separate breakpoints creating the -α ^3.7I^ type, provided one potential explanation for such haplotype diversity. These breakpoints occur within 1kb 5' to the IVS2 region. Across this region, we found very few differences in the human reference sequences and fewer in the control Kenyan sequences, which makes it difficult to determine more precisely where the breakpoints are or how many other breakpoint events may have occurred in the past.

Multiple lines of evidence now point to a model for the occurrence of -α ^3.7I^ deletions, which leads to haplotype patterns that are refractory to imputation. First, it has been observed that -α ^3.7I^ occurs with high mutation rates *in vitro* ^5, 6^. Second, compatible with this high mutation rate, haplotypes carrying -α ^3.7I^ deletions occur across the genealogy of our sample of 3,036 individuals. Third, there is evidence from heterozygous yeast and *Arabidopsis* cells that large (from 100's bp to several kb) insertions/deletions can force the formation of loops of the non-paired DNA that can result in aberrant recombination and new haplotypes ^44, 45^. Further supporting this, we have directly observed evidence that segregating -α ^3.7I^ deletions vary in the locations of their deletion breakpoints by up to 1kb. Given that the -α ^3.7I^ carrying haplotypes are spread throughout the haplotype tree, this situation is particularly challenging for LD-based imputation approaches, which rely on matching of haplotypes at flanking genetic variants to infer the presence of mutations. The similarity between carrier and non-carrier haplotypes across the tree suggests this may not be easily solvable, even with data from additional reference panels.

Notwithstanding the fact that in agreement with observations from a recent study conducted in Tanzania ^2^, the sequences identified the Type I -α ^3.7^ deletion event in all the -α/-α individuals samples investigated, we found no high LD signals with SNPs in the flanking regions. This makes detection of the α-thalassaemia signal by use of standard GWAS chip data difficult, and probably explains why despite finding a strong signal of association between malaria and α-thalassaemia when typed directly, we found no similar signals at any of the SNPs in the surrounding genetic region.

As a result of this initial conclusion, and knowing the location, type, and extent of the deletion, we next investigated a range of alternative approaches to describe and detect the α-thalassaemia deletion using our microarray intensity data, that might be helpful in both measuring and correcting for its effects in African GWAS studies. We note that a number of methodologies exist to detect such features genomewide *de novo* and that have been used to impute INDELS into reference panels. Moreover, this was commented on in the supplementary data of a previous study ^3^ where the 1000 genomes imputation reference panel was used (the -α ^3.7^ deletion is imputed as panel ID: EM_DL_DEL34404), in which the authors found a reduced confidence in predicting the deletion genotypes. Thus, rather than provide an exhaustive comparison of methods, we focussed on methodologies directly using the data for features known to be within the deletion sequence or using the genetic structure of the population. Beginning with imputation and given the low LD within the region, we were somewhat surprised to find that we were able to predict the -α ^3.7I ^deletion with positive-predictive values that ranged from 79–94%, depending on the genotype and statistical model. This was sufficient to allow us to detect an overall signal of association with severe malaria that was similar to that seen using direct genotyping, although the signal detected was considerably weaker, with p-values typically being between 100 and 1000 less significant than those derived from direct genotyping. To see if we could improve on these predictions, we next investigated a range of alternative approaches to typing based on GWAS SNP-chip-intensity data, with a focus on six SNPs from within the -α ^3.7I^ deletion. We speculated that the intensity readings for such SNPs would be reduced in samples from affected individuals in a dose-dependent manner, being most marked in homozygotes. While in practice, we found that there was a large degree of overlap in these distributions between individuals of different genotype classes, and that as a consequence the intensity distributions were insufficiently distinct to allow for accurate prediction when interpreted without reference to gold-standard genotype data. Although the intensity distributions were relatively distinct among homozygotes, we found a particularly high degree of overlap in the values of αα/αα and -α/αα subjects, making it difficult to cleanly separate these genotypic groups on the basis of intensity values alone. We therefore attempted to improve our predictions using a variety of model-based approaches. Some of these models required a training set consisting of a small number of directly genotyped samples. Surprisingly we found that 100 samples was sufficient to train such models, potentially making it easier to provide these data for GWAS datasets. Nevertheless, while these approaches seemed encouraging, allowing us to predict the various α-thalassaemia genotypes with sensitivities of 75.6–97.8% and specificities of 85.0–99.2%, the predicted genotypes did not result in consistent signals of association with severe malaria. Although some gave results that were concordant with the true associations, others were highly variable depending on which SNP and model we used. While a number of signals were highly significant, the directions of effect were inconsistent and were often opposite to those derived from direct genotypes (i.e indicating a risk effect rather than protective effect). Moreover, the selection of 100 samples to create the training set was also a factor in the final outcome. These issues mean that including such intensity data to infer α-thalassaemia genotypes in the analysis of GWAS studies would not be helpful. Similarly, although an ever-increasing number of 'relevant' reference panels are now being produced, we would still urge a degree of caution in their use without first determining their usefulness in comparison to direct typing from the target population. To add to this, we have only explored a single form of the -α ^3.7^ deletion in a population where this appears to be the only deletion that is present. In other parts of the world, the -α ^3.7^I deletion occurs together with Type II and Type III deletions to varying degrees. The breakpoints for these alternative deletions are close to but different to that in the Type I. The GWAS chip SNPs within the -α ^3.7^ region may therefore be distributed across the breakpoints depending on the -α ^3.7^ type, giving a more complex intensity pattern. Moreover, there are other α-thalassaemia deletions of varying sizes around the world that can involve one or both of the *HBA* genes ( *HBA1* and *HBA2*). Some of these are common, and some also overlap the -α ^3.7^ deletion region, adding to this complexity. It may be that direct genotyping is therefore still the only solution to understanding the associations between this locus and malaria or other diseases.

## Data availability

### Source data

The Illumina dataset generated for the Kenya samples and analysed during this current study is registered and available at European Genome-phenome Archive under "Genome-wide study of resistance to severe malaria in eleven populations" (EGA study ID EGAS00001001311) with the Kenya data specifically in EGA Data Set: EGAD00010000904. These data have a managed data-access policy. Further details about the study, data and data-access can be found on the MalariaGEN website.

### Underlying data

Harvard Dataverse: Replication Data for: Haplotype Heterogeneity and Low Linkage Disequilibrium Reduce Reliable Prediction of Genotypes for the α ^-3.7I^ form of α-thalassaemia Using Genome-Wide Microarray Data. https://doi.org/10.7910/DVN/YTXAHR.

This project contains the following underlying data:

- Underlying_DATA1.tab (Illumina 2.5MOmni chip features chr16)
- Underlying_DATA2.tab (Raw and analysis data)
- Underlying_DATA3.tab (Raw and analysis data for MRM and CART example runs)
- Underlying_Data4.tab (Mean intensity data per SNP for all individuals in the study for chromosome 16

### Extended data

Harvard Dataverse: Replication Data for: Haplotype Heterogeneity and Low Linkage Disequilibrium Reduce Reliable Prediction of Genotypes for the α ^-3.7I^ form of α-thalassaemia Using Genome-Wide Microarray Data. https://doi.org/10.7910/DVN/YTXAHR.

This project contains the following extended data:

- Extended_Data.pdf (Supplementary information, figures, tables and Sanger sequencing alignments for *HBA1*, *HBA2* and the α ^-3.7kb^ deletion in Kenyan samples)

Data are available under the terms of the Creative Commons Attribution 4.0 International license (CC-BY 4.0).

### Code availability

Reproducibility code available from: https://www.well.ox.ac.uk/~gav/resources/compute.ld.R and an archived version is included in the Extended_Data.pdf file described above.
