# Supplementary material for: Haplotype heterogeneity and low linkage disequilibrium reduce reliable prediction of genotypes for the ‑α 3.7I form of α-thalassaemia using genome-wide microarray data
Source: Wellcome Open Res. 2021 Sep 22;5:287. Originally published 2020 Dec 9. [Version 2] doi: 10.12688/wellcomeopenres.16320.2 (PMC8474104; doi:10.12688/wellcomeopenres.16320.2)
Supplement: Peer review history

# Peer review: Reviewer response for version 2

**DOI:** 10.21956/wellcomeopenres.18983.r45981

**Published:** 2021-09-27

**Authors:** Manit Nuinoon

**Affiliations:** 1 School of Allied Health Sciences, Walailak University, Nakhon Si Thammarat, Thailand

All my comments have been addressed in the revised manuscript and the authors clearly support the supplementary results of all issues.

Is the work clearly and accurately presented and does it cite the current literature?

Yes

If applicable, is the statistical analysis and its interpretation appropriate?

I cannot comment. A qualified statistician is required.

Are all the source data underlying the results available to ensure full reproducibility?

Yes

Is the study design appropriate and is the work technically sound?

Yes

Are the conclusions drawn adequately supported by the results?

Yes

Are sufficient details of methods and analysis provided to allow replication by others?

Yes

Reviewer Expertise:

Thalassaemia and hemoglobinopathies

I confirm that I have read this submission and believe that I have an appropriate level of expertise to confirm that it is of an acceptable scientific standard.

# Peer review: Reviewer response for version 2

**DOI:** 10.21956/wellcomeopenres.18983.r45982

**Published:** 2021-09-23

**Authors:** Karen G Scheps

**Affiliations:** 1 Departamento de Microbiología, Inmunología, Biotecnología y Genética, Cátedra de Genética, Facultad de Farmacia y Bioquímica, Universidad de Buenos Aires, Buenos Aires, Argentina; 2 CONICET, Buenos Aires, Argentina

All the previous questions have been properly answered and have been addressed in the manuscript.

Is the work clearly and accurately presented and does it cite the current literature?

Yes

If applicable, is the statistical analysis and its interpretation appropriate?

Yes

Are all the source data underlying the results available to ensure full reproducibility?

Yes

Is the study design appropriate and is the work technically sound?

Yes

Are the conclusions drawn adequately supported by the results?

Yes

Are sufficient details of methods and analysis provided to allow replication by others?

Yes

Reviewer Expertise:

Genetics of hemoglobinopathies

I confirm that I have read this submission and believe that I have an appropriate level of expertise to confirm that it is of an acceptable scientific standard.

# Peer review: Reviewer response for version 1

**DOI:** 10.21956/wellcomeopenres.17939.r44833

**Published:** 2021-07-28

**Authors:** Manit Nuinoon

**Affiliations:** 1 School of Allied Health Sciences, Walailak University, Nakhon Si Thammarat, Thailand

The authors applied SNP genotypes spanning 400 kb of the entire a-globin gene cluster region from GWAS data for imputing 3.7 kb type 1 deletion (α-thalassaemia 2). The GWAS chip SNPs within the -α ^3.7I^ deletion region distributed across the breakpoints resulting in a more complex intensity pattern and low linkage disequilibrium (LD). Therefore, it is challenging to apply GWAS chip SNPs for imputing α-thalassaemia. The authors used Sanger sequencing as direct genotyping (reference method) to confirm the form of α-thalassaemia and SNP genotype spanning around -α ^3.7I^ deletion region. Several models were constructed to predict α-thalassaemia genotypes according to single SNP or combined SNPs resulting in different predictive performances. This paper is well written and addresses the detail of each step, and the study design was apparent. My suggestions are as follows:

1. According to the MRM and CART models (Table 6), is it possible to select the same ethnicity for calculating the predictive performance?
2. ROC curve (AUC calculation) is recommended for depicting the predictive performance of single SNP or combined SNPs.
3. Regarding the α-globin gene cluster is located close to the telomere, please put more information about how the telomeric region of this gene cluster affects a more complex intensity pattern in the discussion part.
4. Regarding the main text and several figures, two formats of the 3.7 kb type 1 deletion are found in this article (-α ^3.7I^ deletion and α ^-3.7I^ deletion). Would you please use the same format throughout the manuscript (commonly-used format is -α ^3.7I^ deletion)?
5. According to the title of Table 3, the number of normal individuals should be changed from 5 to 9?

Is the work clearly and accurately presented and does it cite the current literature?

Yes

If applicable, is the statistical analysis and its interpretation appropriate?

I cannot comment. A qualified statistician is required.

Are all the source data underlying the results available to ensure full reproducibility?

Yes

Is the study design appropriate and is the work technically sound?

Yes

Are the conclusions drawn adequately supported by the results?

Yes

Are sufficient details of methods and analysis provided to allow replication by others?

Yes

Reviewer Expertise:

Thalassaemia and hemoglobinopathies

I confirm that I have read this submission and believe that I have an appropriate level of expertise to confirm that it is of an acceptable scientific standard.

# Response

**Published:** 2021-08-26

**Authors:** Kirk Rockett

**Affiliations:** Univerity of Oxford, UK

We thank the reviewers' for their positive and helpful feedback and are pleased to make revisions accordingly as per our comments to their questions below.

 We have therefore added further detail to our manuscript with positional references to the published PDF version.

**1.            According to the MRM and CART models (Table 6), is it possible to select the same ethnicity for calculating the predictive performance?**

 Thank you.  We did not separate the different ethnicities for the MRM and CART as these methodologies rely on the intensity pattern of the SNP features rather than the surrounding genetic structure and therefore should not be affected by a person's origin (unless it is sample related in some way).  Rather the data should be affected more by experimental methodology, reagents, samples and the microarray.  Moreover, one has to balance this with the situation where some ethnic groups may have insufficient numbers to make any methodology viable, particularly when using the data to generate a reference panel as well (as in our group labelled OTHER which is a mixture of 18 different self-reported groups each with too few individuals to ethically identify them or provide sufficient power alone.  Obviously one has to review the results with that in mind). 

 However for this very reason, amongst others, we have made all the data available to allow it to be tested by interested parties.

 This comment also hints at the main problem with microarray data and that is its quality, whether due to the specificity of the individual array features (for example the feature for SNP rs2541670 [Extended Data section 4] and main text figure 4) may behave differently in WT chromosomes versus deletion chromosomes, or sample quality (this data set did have 762 sample excluded for various reasons during its initial QC [see methods]) and even the distribution of SNP intensities across the genome that can affect the genotype calling confidence (as alluded to in point 3 below and in 3 new added figures). 

**2.     ROC curve (AUC calculation) is recommended for depicting the predictive performance of single SNP or combined SNPs.**

 Thank you and all this data is included in the Extended Data file and data package.

**3.   Regarding the α-globin gene cluster is located close to the telomere, please put more information about how the telomeric region of this gene cluster affects a more complex intensity pattern in the discussion part.**

 Thank you and this is clearly an omission on our part.  We have now provided new figures in the Main and Extended Data (Main Figure 11 and Extended Data Figures 12-14) along with accompanying text in both the Main Text and Extended Data text.

***Main Text: page 18, section; 'α-thalassaemia genotype was correlated with intensity signals for SNPs within the -α3.7I deletion'***

*"Given that we were unable to predict α-thalassaemia genotypes with sufficient reliability through the use of LD or imputation methods, we next investigated the potential for an alternative approach – the use of intensity data at SNPs that lie within the deleted region (Underlying Data2 Table OO_intensity_summary).   For the purpose of these analyses, we therefore summed the chip channel intensities (X + Y) to produce a single total intensity value for each individual across each SNP across chromosome 16. We first plotted these intensities averaged over 100kb bins for the whole chromosome (Figure 11A) showing that there was an end-of-chromosome reduction in intensities (from ~ 1.25 to ~ 0.75). At the individual SNP level the mean intensity for the 0-1Mb 5' telomere region where the HBA region lies (Figure 11B) was ~0.75-1.*

*NEW FIGURE 11 showing the SNPs intensities across the full chromosome and within the 5' telomere and HBA regions.*

*The HBA region lies between 200 and 235kb from the 5' telomeric end where the mean intensities were ~ 0.75 (Figure 11B and C, and Extended Data Figure 12-14). While this may be sufficient to have some impact on the predicative performance of the data we* *We hypothesized that intensity signals for such SNPs within the deletion region would still be reduced in α-thalassaemic subjects and that reductions would be dose-dependent in such a way that they would be greatest in homozygotes. We identified six features (rs2362744, rs2854120, rs4021971, rs4021965, rs11639532 and rs2858942) on the Illumina HumanOmni2.5-4 chip that lay within the -α ^3.7I^ deletion, along with three flanking SNPs that served as controls (Figure 12 and Underlying Data2 Table OO_intensity_ summary). For the purpose of these analyses, we summed the chip channel intensities (X + Y) to produce a single total intensity value for each individual at wach of the six SNPs within the deletion and three flanking SNPs. These Sum(X+Y) data for each individual and each of the 6 SNPs data were then plotted as means with 25 th and 75 th percentiles and outliers on the y-axis with stratification by α-thalassaemia genotype on the x-axis (Figure 12, Extended Data Figures 16 and as scatter plots Extended Data Figure 17). As anticipated, we saw significant step-wise reductions in intensities by α-thalassaemia genotype for all six features within the deletion (Kruskall-Wallis and Dunn's test for multiple comparisons ^21^ ; Extended Data Section 4, Underlying Data2 Tables PP_intensity_comparisons and QQ_intensity_summary), leading us to investigate a range of potential methods for predicting α-thalassaemia genotypes from such data."*

**Extended Data Section 4, new introductory paragraph;** **Illumina Chip Intensities**

*"We first extracted the intensities for all features from the chromosome 16 vcf file for the 3036 samples used in this study and then plotted the mean of the Sum(X+Y) channels for these samples for each SNP against chromosomal position (Figure 12A).   This showed that the feature average intensities across the chromosome varied from ~0.25 to 2.  In the smoothed plot and 100kb windowed plots (Figure 12B, C and D) it can be seen that the general intensity across the chromosome is ~1.25 while at the telomeres (start-10Mb and 85Mb to end), the intensities drop gradually to ~0.75 and 1 respectively.  This is not unusual for such microarray data as was noted in the MalariaGEN study ^3 ^that generated this GWAS data.  Focussing in on the region of interest, Figure 13 shows the first 1Mb region at the 5' telomere and Figure 14 the region across the HBA gene cluster (200kb to 235kb).  These plots show the variation between SNP intensities, which are all reduced (~0.75) in comparison with the central region (~1.0) of the chromosome intensities.  Notwithstanding this, it is important to review the distribution of sample intensities for each SNP."*

**4.     Regarding the main text and several figures, two formats of the 3.7 kb type 1 deletion are found in this article (-α ^3.7I^ deletion and α ^-3.7I^ deletion). Would you please use the same format throughout the manuscript (commonly-used format is -a ^3.7I^ deletion)?**

 Thank you and we have checked, corrected, and standardised throughout the manuscript depending on the context used.  When referring to the 3.7kb deletion collectively or where the type is unknown we use -α ^3.7^, while the suffix I is added when specifically referring to the Type I variant (and similarly for Type II and Type III).

 These have been corrected accordingly through both the Main text and Extended_Data text.

**5.     According to the title of Table 3, the number of normal individuals should be changed from 5 to 9?**

 Thank you, this has been done.

# Peer review: Reviewer response for version 1

**DOI:** 10.21956/wellcomeopenres.17939.r44831

**Published:** 2021-07-20

**Authors:** Karen G Scheps

**Affiliations:** 1 Departamento de Microbiología, Inmunología, Biotecnología y Genética, Cátedra de Genética, Facultad de Farmacia y Bioquímica, Universidad de Buenos Aires, Buenos Aires, Argentina; 2 CONICET, Buenos Aires, Argentina

GWAS studies are a powerful tool to contribute to the knowledge of the bases of common and rare diseases. However, many studies do not contemplate the effects of CNVs, which could bias the obtained results. This matter is particularly relevant when studying the association of risk of severe malaria in African populations since α-thalassemia (thal) mutations are prevalent and the long ancestral history of this group has led to low linkage disequilibrium.

 The authors proposed a very straightforward analysis flow to infer α-thal genotypes based on the data obtained by GWAS –based approaches. The design was very clear and the partial results obtained in each analysis provide valuable information for the field since it could potentially be applied for retrospective analysis of other GWAS-obtained data.

 I have a few questions for the authors and I believe that the answers could be included in the manuscript as well.

  

1. Could it be possible to analyze the GWAS-obtained data investigated in this study with any of the platforms developed for genomic profiling of CNVs, such as Beadstudio or PennCNV?
2. For the approach of imputation using haplotypes, it is understandable why 1000G reference panel should not be used. Would you consider using the African/African-American genomes included in gnomAD v3.1?
3. Regarding the Chip Intensity (sum[X and Y] channels) density plots featured in Figure 4, is it possible that the lack of troughs or shoulders for SNPs rs4021965 and rs2858942, altered the chances of discriminating the different α-thalassemia (thal) genotypes with this approach? When analyzing the intensity of these SNPs in the different  α-thalassemia (thal) genotypes it would seem that at least the homozygous state could be discriminated

Is the work clearly and accurately presented and does it cite the current literature?

Yes

If applicable, is the statistical analysis and its interpretation appropriate?

Yes

Are all the source data underlying the results available to ensure full reproducibility?

Yes

Is the study design appropriate and is the work technically sound?

Yes

Are the conclusions drawn adequately supported by the results?

Yes

Are sufficient details of methods and analysis provided to allow replication by others?

Yes

Reviewer Expertise:

Genetics of hemoglobinopathies

I confirm that I have read this submission and believe that I have an appropriate level of expertise to confirm that it is of an acceptable scientific standard, however I have significant reservations, as outlined above.

# Response

**Published:** 2021-08-26

**Authors:** Kirk Rockett

**Affiliations:** Univerity of Oxford, UK

We thank the reviewers' for their positive and helpful feedback and are pleased to make revisions accordingly as per our comments to their questions below.

 We have therefore added further detail to our manuscript with positional references to the published PDF version.

**1.      Could it be possible to analyze the GWAS-obtained data investigated in this study with any of the platforms developed for genomic profiling of CNVs, such as Beadstudio or PennCNV?**

 Thank you for this suggestion.  This was certainly something that we had considered but we wanted to make the main focus of the manuscript about the haplotype structure and association with malaria rather than an exhaustive comparison of methodologies.  Our study was therefore not designed to try and compare and contrast all available methods, nor identify any failings or provide suggestions for correcting them, but rather provide a description of the intensity data in the region and along with haplotype structure try and provide some explanation why prediction/imputation methods may struggle to properly call the alpha-thalassaemia locus, and therefore drawing the wrong conclusions about the effect and significance in malaria.  We do provide details of where all the relevant data can be found for interested parties to test these methods of their own designs.

 We also note that this region has already been commented on using the data from the 1000 genomes study where such methods were used to call the alpha-thalassaemia locus in several populations with similar findings to our current manuscript (Malaria Genomic_Epidemiology_Network, Band G, Quang SL, et al. (2019) Insights into malaria  susceptibility using genome-wide data on 17,000 individuals from Africa, Asia and Oceania. Nat Commun.; 10: 5732).

**Main Text Page 6, new introductory paragraph for section; 'Inferring α-thalassaemia genotypes from intensity data'**

*"Given that we know the location and breakpoints for the* *a* *-thalassaemia deletion under investigation, we have looked at the SNPs and intensities directly to determine in an a priori fashion how well they infer the genotypes for the -* *a* *^3.7^ deletion.  We note that other predicative/inference methods are available in addition to those we have used, some designed to identify/discover non-SNP features using intensity data. Accordingly, we have provided all the information and data necessary for others to test alternative methodologies if interested.* *"*

**Main Text Page 23, Discussion paragraph 5; we have modified and added a section at the beginning of this paragraph to comment on our choice of methodologies for looking at the imputation of genotype from intensity data.**

*"As a result of this initial conclusion, and knowing the location, type, and extent of the deletion, we next investigated a range of alternative approaches to describe and detect the α-thalassaemia deletion using our microarray intensity data, that might be helpful in both measuring and correcting for its effects in African GWAS studies. We note that a number of methodologies exist to detect such features genomewide *de novo *and that have been used to impute INDELS into reference panels. Moreover, this was commented on in the supplementary data of a previous study ^3^ where the 1000 genomes imputation reference panel was used (the -α ^3·7^ deletion is imputed as panel ID: EM_DL_DEL34404), in which the authors found a reduced confidence in predicting the deletion genotypes.   Thus, rather than provide an exhaustive comparison of methods, we focussed on methodologies directly using the data for features known to be within the deletion sequence or using the genetic structure of the our population. "*

**2.            For the approach of imputation using haplotypes, it is understandable why 1000G reference panel should not be used. Would you consider using the African/African-American genomes included in gnomAD v3.1?**

 We agree that other reference panels may be more appropriate to use and we did allude to this in our text, but have now made this clearer.  However, through our work with MalariaGEN we note that even that may not be sufficient. Furthermore from our data where we are using the very same population and data as both reference panel and test data, the predictions/imputations were still not sufficient to provide complete confidence in the analyses compared with using directly typed data.  We have added to the comments in our discussion on this point (see point 1 above), and to the methods section. However we have made all the data available and therefore this can be tested given an individual's data and circumstances.  But our results do suggest caution in that even with some level of correctly predicted/imputed genotypes an incorrect outcome may result with a high level of significance leading to spurious conclusions.

**Main Text Page 13, and Extended Data page 52, section; 'Imputation using Haplotypes'**

*"IMPUTE2 requires a reference set of haplotypes to infer missing genotypes. However, we decided not to use the 1000 genome reference panel for several reasons;*

- *The 1000G dataset does not contain any directly typed α-thalassaemia deletion variants, only imputed from the sequence data.*
- *The 1000G dataset does not have any populations that directly match the populations used in our study (the closest is a Western Kenyan population [LWK – Luhya]).*
- *There is also evidence that the 1000G imputed α-thalassaemia genotypes are not very reliable ^3^.*

*Other reference panels are becoming more widely available, some of which also include additional populations of African origin. While we decided not to use these for similar reasons to those described above for the 1000 Genomes data, we do not rule out the possibility that some could prove more suitable for imputing haplotypes into Kenyan populations.*

*Instead we used our own data for creating both a reference dataset and for imputing missing genotypes, as this should have provided the best opportunity to predict/impute the α-thalassaemia genotypes."*

**

**3.     Regarding the Chip Intensity (sum[X and Y] channels) density plots featured in Figure 4, is it possible that the lack of troughs or shoulders for SNPs rs4021965 and rs2858942, altered the chances of discriminating the different α-thalassaemia (thal) genotypes with this approach? When analyzing the intensity of these SNPs in the different  α-thalassaemia (thal) genotypes it would seem that at least the homozygous state could be discriminated**

 Thank you, yes that is exactly what we believe and tried to describe.  We have reviewed our text and re-worded the appropriate text to make these points clearer. 

***Main Text Page 18, section; 'Signal intensities alone were not enough to predict α-thalassaemia genotype with any accuracy'***

*"Based on the assumption that most investigators would not have access to direct genotyping, we first created histogram and density plots of the intensity data from the nine SNPs described above, without reference to data on α-thalassaemia genotype (Figure 4). From these data we identified troughs or shoulders to separate the different genotype classes;   SNPs rs4021965 and rs2858942 did not show a trough or shoulder to help identify heterozygotes from WT normal individuals, although they did show some discrimination of the homozygotes.  But four SNPs (rs2362744, rs2854120, rs4021971, rs11639532) did show possible breaks between each genotype class (Figure 4 and Extended Data Table 3 and Underlying Data2 Table RR_intensity_cutoffs) and we focused on these to infer α-thalassaemia genotypes from these groupings ..."*

# Response

**Published:** 2021-08-26

**Authors:** Kirk Rockett

**Affiliations:** Univerity of Oxford, UK

We thank the reviewers' for their positive and helpful feedback and are pleased to make revisions accordingly as per our comments to their questions below.

 We have therefore added further detail to our manuscript with positional references to the published PDF version).

 My apologies but this is an additonal revision based on reviewer #1's comment #2 ommitted from my original post.

**2.            For the approach of imputation using haplotypes, it is understandable why 1000G reference panel should not be used. Would you consider using the African/African-American genomes included in gnomAD v3.1?**

**Main Text Page 25, Final section of the discussion.**

 "... *Similarly, although an ever increasing number of 'relevant' reference panels are now being produced, we would still urge a degree of caution in their use without first determining their usefulness in comparison to direct typing from the target population.  To add to this, we have only explored a single form of the -α ^3.7^ deletion in a population where this appears to be the only deletion that is present. In other parts of the world, the -α ^3.7I^ deletion occurs together with Type II and Type III deletions to varying degrees. The breakpoints for these alternative deletions are close to but different to that in the Type I.  The GWAS chip SNPs within the -α ^3.7^ region may therefore be distributed across the breakpoints depending on the -α ^3.7^ type, giving a more complex intensity pattern. Moreover, there are other α-thalassaemia deletions of varying sizes around the world that can involve one or both of the HBA genes (HBA1 and HBA2). Some of these are common, and some also overlap the -α ^3.7^ deletion region, adding to this complexity.  It may be that direct genotyping is therefore still the only solution to understanding the associations between this locus and malaria or other diseases."*
